# ^68^Ga-Labeled TRAP-Based Glycoside Trimers for Imaging of the Functional Liver Reserve

**DOI:** 10.1021/acs.jmedchem.4c02006

**Published:** 2024-10-16

**Authors:** Maximilian A. Zierke, Christine Rangger, Kimia Samadikhah, Christoph Kreutz, Andreas M. Schmid, Roland Haubner

**Affiliations:** † Department of Nuclear Medicine, Medical University of Innsbruck, Anichstr. 35, 6020 Innsbruck, Austria; ‡ Werner Siemens Imaging Center, Department of Preclinical Imaging and Radiopharmacy, Eberhard Karls University Tübingen, Röntgenweg 13, 73076 Tübingen, Germany; § Institute of Organic Chemistry and Center for Molecular Biosciences (CMBI), University of Innsbruck, Innrain 80–82, 6020 Innsbruck, Austria

## Abstract

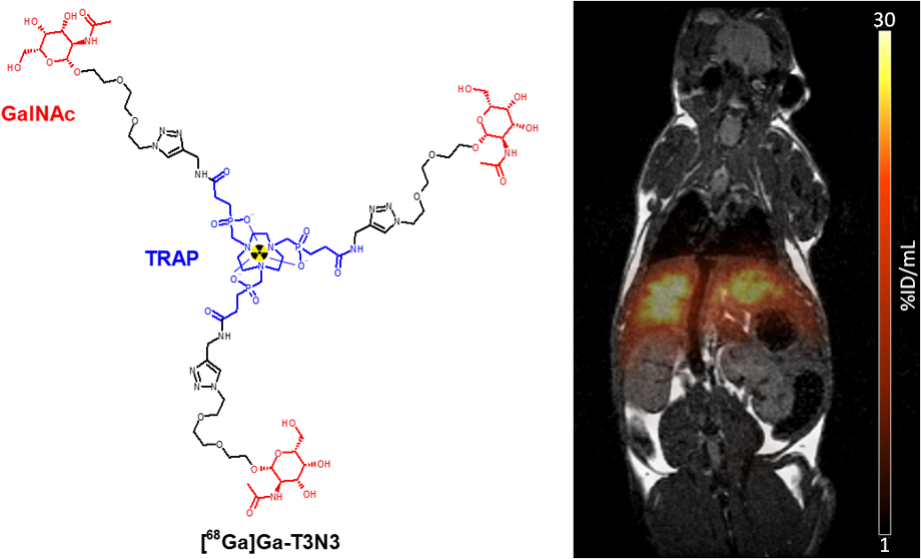

The exclusive asialoglycoprotein receptor (ASGR) expression on hepatocytes makes it an attractive target for imaging of the functional liver reserve. Here, we present a set of TRAP-based glycoside trimers and evaluate their imaging properties compared to the gold standard [^99m^Tc]Tc-GSA. The click-chemistry-based synthesis approach provided easy access to trimeric low-molecular-weight compounds. Labeling with ^68^Ga was carried out in high radiochemical yields (>99%). Complexes showed high stability and hydrophilicity. Protein binding ranged between 10 and 25%. Highest binding affinity (IC_50_) and best liver accumulation were found for [^68^Ga]Ga-**T3N3**, followed by [^68^Ga]Ga-**T3G3** and [^68^Ga]Ga-**T0G3**. Rapid elimination from the rest of the body resulted in excellent target-to-background ratios. Our studies confirmed that high ASGR uptake depends on the correct spacer design and that *N*-acetylgalactosamine improves targeting properties *in vivo*. Thus, [^68^Ga]Ga-**T3N3** represents a new low-molecular-weight radiopharmaceutical with pharmacokinetics similar to those of [^99m^Tc]Tc-GSA.

## Introduction

Over the past few years, multimeric ligand concepts have gained increased popularity in the field of nuclear medicine as they can have a bunch of favorable properties such as increased hydrophilicity, improved stability, and elevated affinity compared to the monomeric conjugate.^1−3^ However, a robust prediction of the behavior of these compounds based solely on the chemical structure stays rather vague. Lately, synergistic interactions have been introduced to serve as an explanation for their altered *in vivo* behavior.^4^ Nevertheless, some target structures may require the presence of multiple possible binding sites within the ligand to allow for high-affinity interactions.^5,6^

One such target is the asialoglycoprotein receptor (ASGR), a 140 kDa protein that is almost exclusively found on the apical side of functional hepatocytes.^7,8^ The receptor is a c-type lectin comprising two H1 and one H2 subunit, each bearing a carbohydrate recognition domain.^9^ Its physiological function is the removal of desialylated glycoproteins upon ligand binding and internalization via clathrin-coated pits.^10^ As it has been shown that ASGR expression correlates inversely with the progression of various liver-specific diseases such as NAFLD, NASH, cirrhosis, and hepatocarcinoma, functional liver imaging addressing the ASGR status has gained increased value for disease monitoring.^11−13^ Furthermore, preoperative assessment of remnant liver function is a key factor for patient outcomes in various clinical settings including surgery and transplantation.^14,15^

Due to the trimeric structure of this receptor, it has become quite evident that the most suitable ligands for this target are indeed multimers featuring galactose or *N*-acetylgalactosamine as pharmacophore.^16−20^ Examples for this class of compounds have been summarized in a comprehensive review by Huang et al.^21^ Recently, our group has presented [^68^Ga]Ga-NODAGA-TriGalactan, a small-molecule-based ASGR tracer allowing for functional liver imaging with positron emission tomography (PET).^22^ The ligand design concept of this tracer features TRIS as a branching unit bearing three galactose moieties at an approximate distance of 19 Å. Although this concept allows for a wide range of modifications, including exchange of the chelator by a dye or a drug, synthesis of the organic backbone is tedious and requires several HPLC purification steps. Furthermore, the overall low synthesis yield might be a drawback when it comes to the translation of precursor production to an industry scale. Therefore, alternative pathways for designing small-molecule-based ASGR tracers have been investigated.

TRAP is a phosphinic acid analogue of NOTA developed by Notni et al. for selective complexation of ^68^Ga.^23^ To the best of our knowledge, no ASGR tracer based on this multivalent chelator has been published to date. The main advantage of using TRAP instead of NOTA is the ability to functionalize all three arms of the chelator simultaneously and, hence, generate a functional trimer without losing its ability to stably bind gallium. This methodology has already been applied for the synthesis of a wide range of radioligands for different target structures including tracers for PSMA^24^ and integrins α_5_β_1_,^25^ α_v_β_3_,^26^ α_v_β_6_,^5,27^ and α_v_β_8_.^28^ Therefore, the aim of this study was to generate a small set of TRAP-based glycoside trimers and to investigate their suitability as PET imaging agents for the ASGR.

## Results

### Chemical Synthesis

**Scheme 1:**
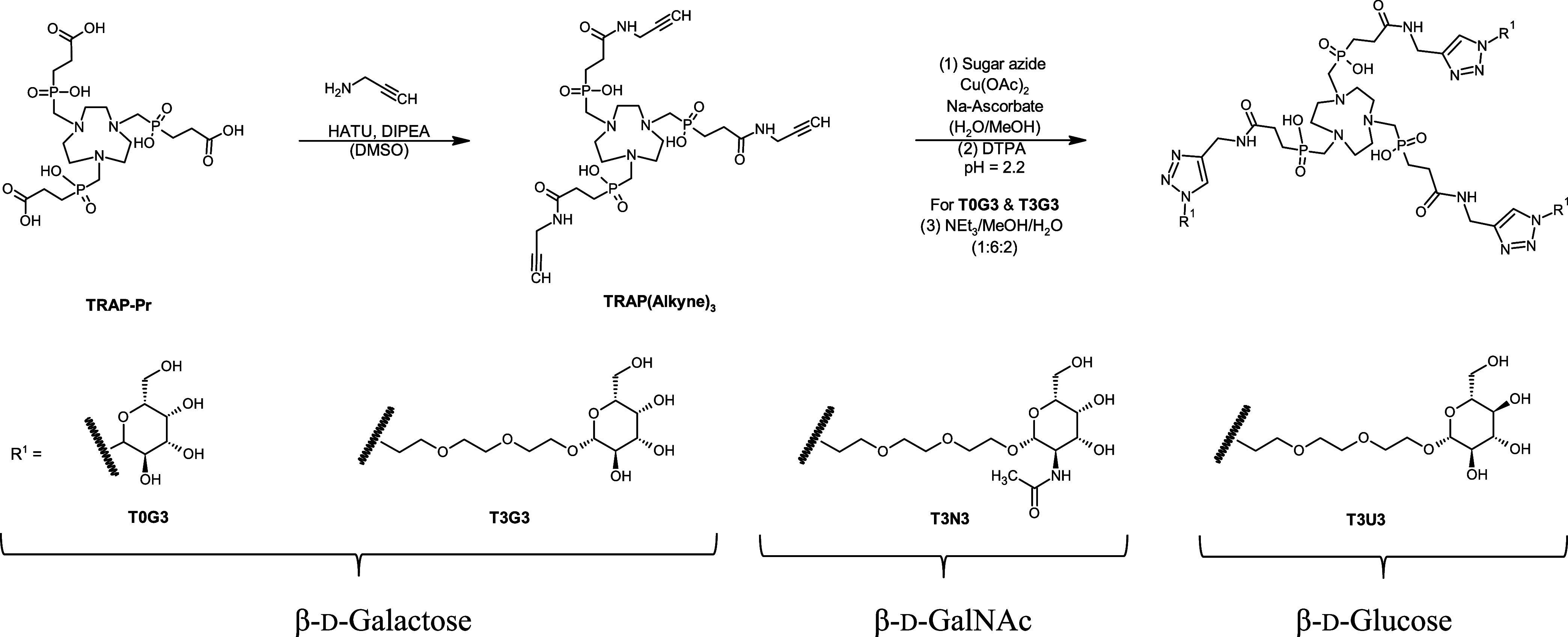
Synthetic Route of the TRAP-Based Glycoside Trimers

Synthesis started with **TRAP-Pr**, which was modified using HATU and propargylamine to obtain **TRAP(Alkyne)**_**3**_ (Scheme 1). Next, copper-catalyzed azide–alkyne cycloaddition was used to attach different sugar azides to the chelator. Selected saccharides were β-d-galactose (**T3G3**), β-d-*N*-acetylgalactosamine **(T3N3**), and β-d-glucose (**T3U3**) each featuring a PEG_3_ linker as a spacer. Glucose was chosen to obtain a negative control compound. To study the influence of the PEG linker, one compound had β-d-galactose directly attached to the TRAP core (**T0G3**). Copper species were removed by *in situ* demetalation and all trimers were purified via semipreparative HPLC. In the case of **T0G3** and **T3G3**, an additional acetyl deprotection step of the galactose units was required. All labeling precursors were obtained in yields ranging from 5–25% (Table 1). Due to metal contaminants, chemical purities of two of the four compounds were found to be slightly below 95%. Mass spectral analysis revealed Fe^3+^ complexes as the most apparent impurity. As radiolabeling properties were not significantly impaired by these findings, further repurification attempts were omitted.

**Table 1:** Analytical Data of TRAP-Based Glycoside Trimers

	monoisotopic mass calcd. (Da)	monoisotopic mass found (*m*/*z*)	*t*_R_ HPLC (min)	purity^c^ (%)	yield^d^ (%)
**T0G3**	1305.46	1305.8 [M + H]^+^	9.0^a^	95	25
**T3G3**	1701.70	1748.1 [M + 2Na + H]^+^	13.5^b^	94	5
**T3N3**	1824.77	1823.8 [M – H]^−^	14.5^b^	96	10
**T3U3**	1701.70	1703.1 [M + H]^−^	13.6^b^	94	13

a Gradient: 1–10% B in 15 min, 1 mL/min, ReproSil Pur C18 AQ.

b Gradient: 5–15% B in 15 min. 1 mL/min, ReproSil Pur C18 AQ

c Determined by HPLC.

d Calculated based on the amount of TRAP(Alkyne)_3_.

### Labeling with ^68^Ga and ^nat^Ga

**Figure 1:**
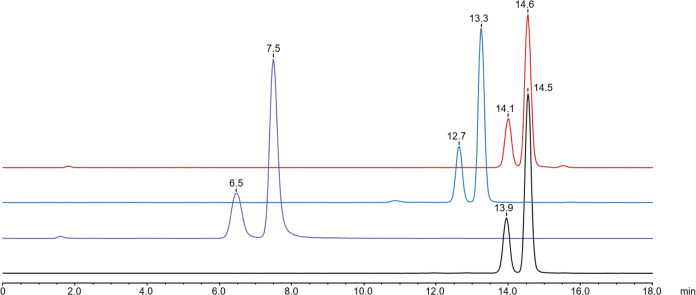
Radio-HPLC chromatograms of ^68^Ga-labeled compounds [^68^Ga]Ga-**T3N3** (red), [^68^Ga]Ga-**T3G3** (blue), [^68^Ga]Ga-**T0G3** (purple), and [^68^Ga]Ga-**T3U3** (black); Column: ReproSil Pur C_18_ AQ, 150 × 4.6 mm^2^, 5 μm, 120 Å; Solvent A: H_2_O/0.1% TFA, Solvent B: MeCN/0.1% TFA; Flow: 1 mL/min; Gradient: 1–10% B in 15 min (**T0G3**); 5–15% B in 15 min (**T3N3, T3G3, and T3U3**).

Incorporation of ^68^Ga (RCY > 99%, RCP > 97%) was accomplished within 15 min in a 5 M HEPES buffer at 95 °C. Molar activities ranged from 10 to 15 MBq/nmol. Labeling resulted in the formation of two distinct isomers in a ratio of 1:3, which could be baseline separated by analytical HPLC (Figure 1). Most surprisingly, this phenomenon also occurred when a 1.5-fold molar excess of nonradioactive gallium (^nat^Ga) was applied. LC-MS analysis of these metal complexes revealed that both species possess the same mass. The reinjection of one species into the HPLC did not result in the formation of two new species, but heating the sample to 60 °C led to a partial formation of the second species over time. Exemplary HPLC traces can be found in the Supporting Information.

### *In Vitro* Evaluation

High complex stabilities in PBS and human blood serum were observed for up to 2 h as determined by radio-HPLC (Table 2). The highest protein binding was found for ^68^Ga-labeled **T3N3**, followed by **T0G3** and **T3G3** after 120 min of incubation. The glucose trimer [^68^Ga]Ga-**T3U3** showed by far the least interaction with serum proteins, as indicated by its decrease from an initial 3.6% of protein binding to 1.1% within 60 min of incubation. log *D* values revealed high hydrophilicity of all tracers with [^68^Ga]Ga-**T3G3** being the most hydrophilic one, followed by [^68^Ga]Ga-**T3N3**, [^68^Ga]Ga-**T0G3**, and [^68^Ga]Ga-**T3U3**. Binding experiments in an isolated receptor-based assay identified [^68^Ga]Ga-**T3G3** and [^68^Ga]Ga-**T3N3** as the most affine compounds for the ASGR. Removal of the PEG linkers led to a significant (*p* < 0.05) decrease in affinity as found for [^68^Ga]Ga-**T0G3**. The exchange of galactose by glucose as the pharmacophore resulted in a complete loss of affinity, as shown for [^68^Ga]Ga-**T3U3**.

**Table 2:** Complex Stability in Human Blood Serum (*n* = 2) and PBS (*n* = 1), Protein Binding (*n* = 3), IC_50_ (*n* = 3), and log *D* (*n* = 6) Values of TRAP-Based Trimers

	complex stability in human blood serum (% intact ligand)				complex stability in PBS (% intact ligand)				protein binding (%)					
min	2	30	60	120	2	30	60	120	2	30	60	120	IC_50_ (nM)^a^	log *D*
[^68^Ga]Ga-**T0G3**	99.8 ± 0.1	99.9 ± 0	99.9 ± 0	99.9 ± 0	99.5	99.3	99.4	99.3	31.7 ± 1.7	20.9 ± 3.8	17.6 ± 4.9	20.5 ± 5.1	17.0 ± 5.9	–4.16 ± 0.09
[^68^Ga]Ga-**T3G3**	97.7 ± 0.3	97.6 ± 0.2	97.6 ± 0.1	97.9 ± 0.1	99.0	98.9	98.7	98.7	8.2 ± 3.4	6.4 ± 1.8	12.0 ± 3.1	11.6 ± 2.2	3.0 ± 4.5	–4.26 ± 0.08
[^68^Ga]Ga-**T3N3**	99.3 ± 0.1	99.7 ± 0.1	99.6 ± 0.2	99.7 ± 0.1	99.9	99.9	99.9	99.9	21.5 ± 1.7	24.3 ± 5.2	18.0 ± 3.8	25.2 ± 5.1	2.7 ± 2.0	–4.22 ± 0.08
[^68^Ga]Ga-**T3U3**	99.9 ± 0	99.9 ± 0	99.9 ± 0	99.8 ± 0	99.7	99.6	99.5	99.4	3.6 ± 0.9	3.3 ± 2.0	1.1 ± 3.2	-	n.d.a.^b^	–3.80 ± 0.08

a Half-maximum inhibitory concentration of [^125^I]I-Asialoorosomucoid binding to the H1 subunit of human recombinant ASGR.

b No detectable affinity.

### *In Vivo* Evaluation

**Figure 2:**
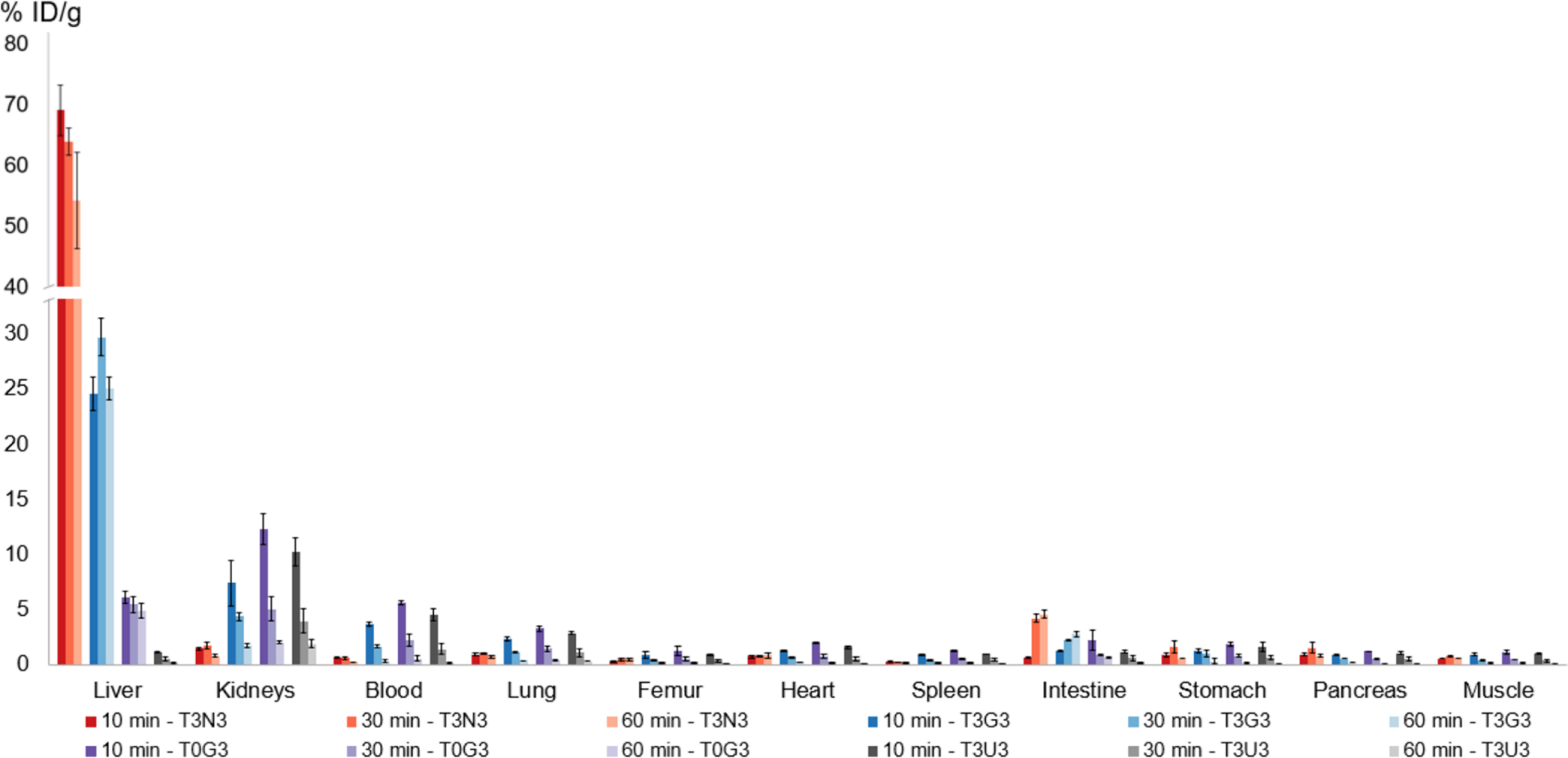
Biodistribution data of TRAP-based glycoside trimers [^68^Ga]Ga-**T3N3**, [^68^Ga]Ga-**T3G3**, [^68^Ga]Ga-**T0G3**, and [^68^Ga]Ga-**T3U3** 10, 30, and 60 min p.i. in healthy female BALB/c mice (100 pmol, 1 MBq).

**Figure 3:**
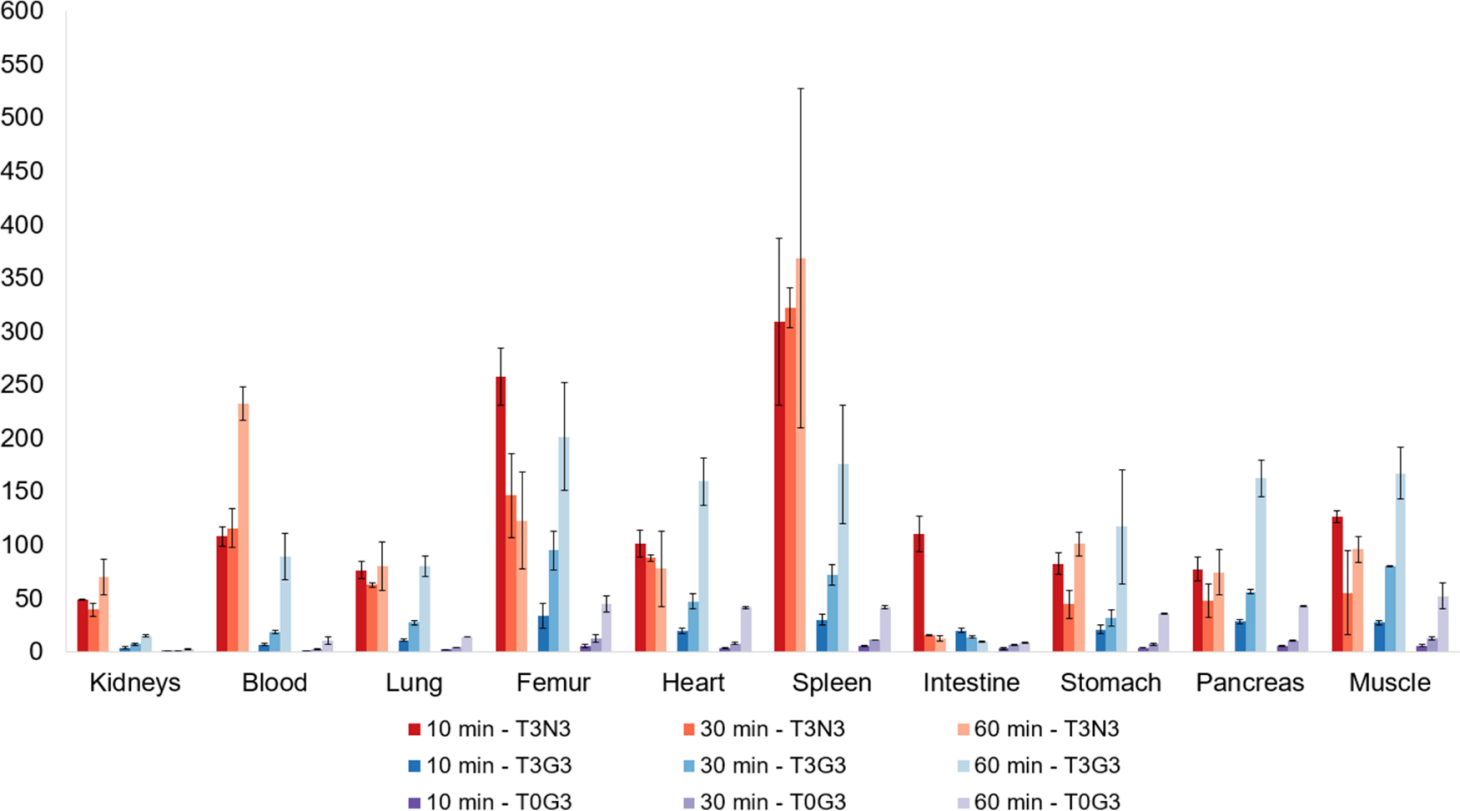
Liver-to-organ ratios for trimers [^68^Ga]Ga-**T3N3**, [^68^Ga]Ga-**T3G3**, and [^68^Ga]Ga-**T0G3** 10, 30, and 60 min p.i. in healthy female BALB/c mice (100 pmol, 1 MBq).

In biodistribution studies, highest liver uptake was found for [^68^Ga]Ga-**T3N3** with negligible off-target uptake in other organs and initially the best liver-to-organ ratios in spleen, femur, muscle, intestine, blood, and heart 10 min p.i. (Figures 2 and 3). Liver uptake stayed at an overall high level and decreased to 54.2 ± 8.0% ID/g 60 min p.i. In contrast, accumulation in the intestine reached its maximum 60 min p.i. and arose from the initial 0.6 ± 0.1% ID/g to 4.5 ± 0.4% ID/g. Blood levels stayed low over the course of the experiment, indicating fast target binding and rapid renal elimination of the unbound ligand. The trimer [^68^Ga]Ga-**T3G3** showed second-best liver accumulation with 29.7 ± 1.7% ID/g peaking at 30 min p.i. Initial uptake was also found in the kidneys and blood pool, followed by a rapid washout from nontarget tissue. In contrast to [^68^Ga]Ga-**T3N3**, where excellent liver-to-organ ratios can already be found at the earliest time point, best liver-to-organ ratios can be found for [^68^Ga]Ga-**T3G3** 60 min p.i. in femur, spleen, muscle, pancreas, heart, stomach, and blood. Removal of all PEG_3_ linkers in [^68^Ga]Ga-**T0G3** resulted in reduced liver and elevated kidney uptake, probably due to enhanced renal excretion of the unbound ligand. As expected and in contrast to all three other compounds, the least liver uptake was found for the negative control compound [^68^Ga]Ga-**T3U3**. However, all four tracers showed minimal uptake in the spleen, pancreas, stomach, heart, lung, muscle, and bone.

**Figure 4:**
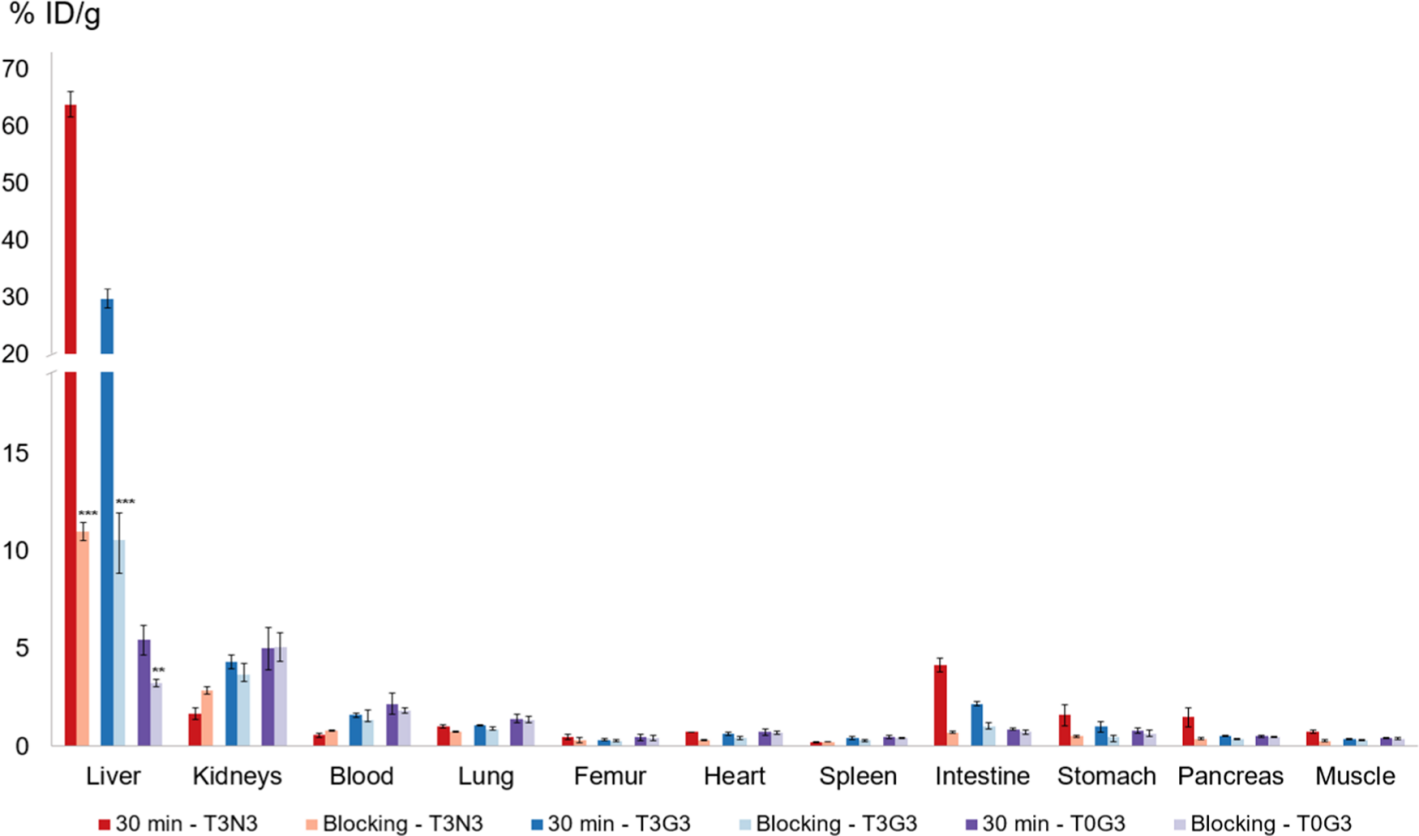
Blocking data of TRAP-based glycoside trimers [^68^Ga]Ga-**T3N3**, [^68^Ga]Ga-**T3G3**, and [^68^Ga]Ga-**T0G3** 30 min p.i. in healthy female BALB/c mice (100 pmol, 1 MBq). For blocking studies, 27.7 μmol of either *N*-acetylagalctosamine (**T3N3**) or d-galactose (**T3G3**, **T0G3**) was co-injected (***p ≤ 0.001; **p ≤ 0.01).

In the blocking experiment, co-injection of *N*-acetylgalactosamine in excess led to a statistically significant reduction of liver uptake for [^68^Ga]Ga-**T3N3** by 83% (*p* ≤ 0.001). Interestingly, further reduced uptake was also observed in the intestine, stomach, and pancreas (Figure 4). When coadministering 27.7 μmol of d-galactose, [^68^Ga]Ga-**T0G3** and [^68^Ga]Ga-**T3G3** showed reduced liver accumulation by 36% (*p* ≤ 0.01) and 60% (*p* ≤ 0.001), respectively. As [^68^Ga]Ga-**T3U3** did not show any significant liver uptake, it was not considered for the blocking experiment.

### *In Vivo* Imaging

**Figure 5:**
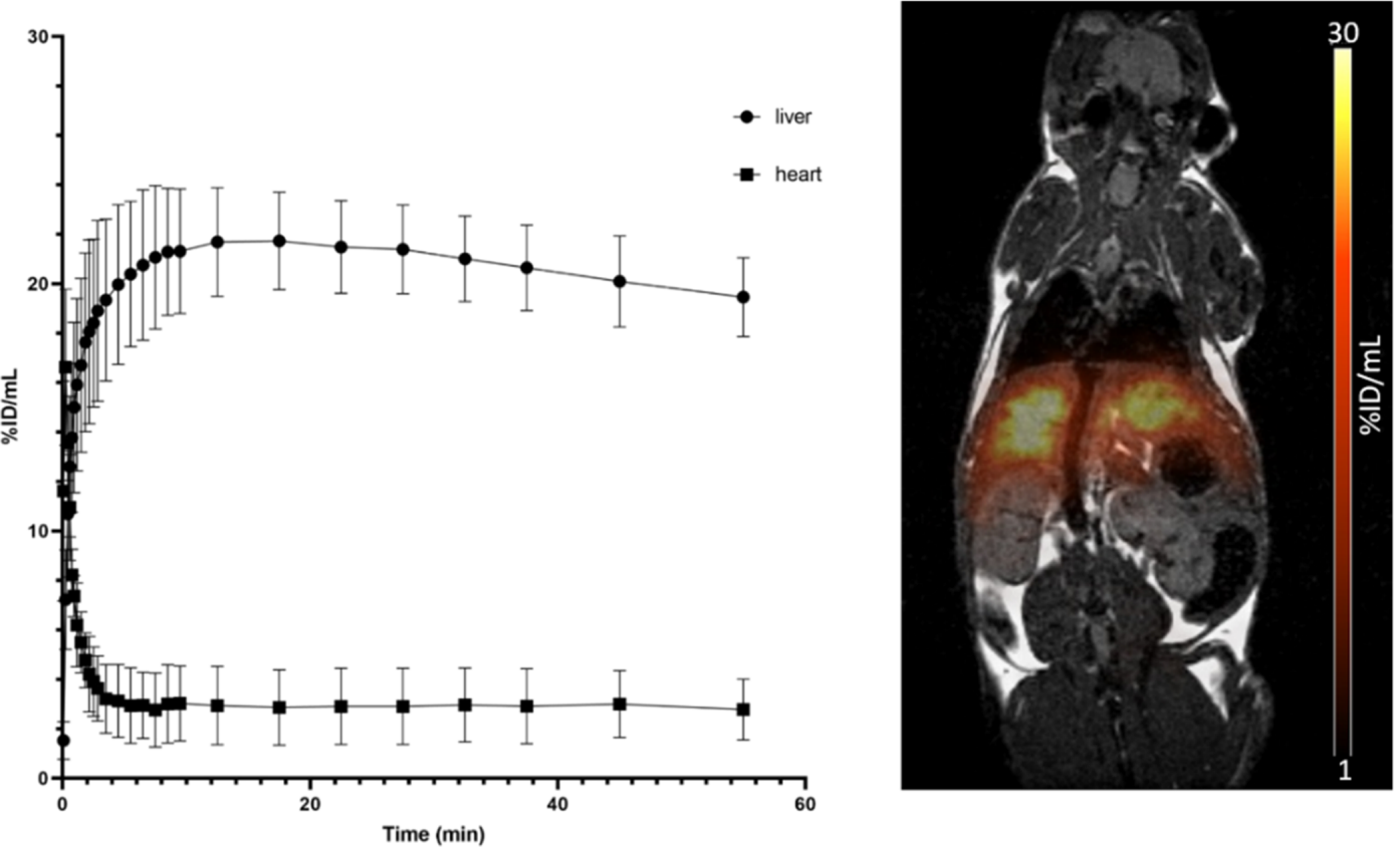
TACS of liver and heart (left) and PET/MR fusion (right) of a healthy male C57BL/6J mouse, injected with 1 MBq (100 pmol) of [^68^Ga]Ga-**T3N3**.

The best candidate ([^68^Ga]Ga-**T3N3)** according to the biodistribution experiments was injected into healthy male C57BL6 and studied with PET/MR (*n* = 3). After injection of the tracer, liver accumulation reached its maximum 10 min p.i., followed by a slight washout over the course of 60 min (Figure 5). Simultaneously, a rapid elimination from the blood pool led to an excellent separation of the time activity curves (TACs) from 5 min p.i. until the end of the observation period. Reconstruction of PET and MR data resulted in a high contrast image allowing a clear delineation of the functional liver tissue.

## Discussion

In this study, we developed trimeric carbohydrates for the determination of ASGR expression using PET imaging. The implementation of TRAP as the branching unit drastically shortened the synthetic procedure. Synthesis of all 4 trimers was generally achieved within 3–4 steps, compared to 7 steps for NODAGA-TriGalactan, our first-generation compound.^22^ As a result, synthesis yields of all trimers were higher than those for NODAGA-TriGalactan (3.2%). In addition, the TRAP platform allowed a rather quick introduction of structural changes regarding sugar type and linker lengths, thus making it an attractive tool for the generation of trimer libraries.

Mass spectral analysis revealed that Fe^3+^ complexes were the most abundant impurity found. Due to the similar ionic radius of Fe^3+^ (0.69 Å) and Ga^3+^ (0.76 Å), both cations can form stable complexes with the TRAP chelator.^23^ However, labeling of the compounds with ^68^Ga was not impaired and resulted in the formation of two distinct conformational isomers as assessed via radio-HPLC. A similar phenomenon has already been described for NOTA-conjugated octreotide labeled with [^18^F]F-AlF.^29^ In the latter case, the presence of two radioactive species was due to the formation of the stereoisomers upon chelation of the pseudometal. It is known that the complexation of gallium induces chiral centers at the phosphorus atoms in the TRAP core.^23^ Theoretically, a total set of four diastereomeric complex geometries is possible.^30^ In fact, X-ray crystallography and NMR studies of Ga^3+^-TRAP complexes confirmed the existence of a nonfluxional pair of enantiomers. This would be in line with our finding that the two species observed here do not readily interconvert at room temperature but require heating. Assuming that the presence of metal-induced conformation isomers does not have a significant influence on ASGR-targeting properties, isolation and separate examination of the conformers were evaded.

The first trimers tested were **T3G3** and **T0G3**, both featuring galactose with or without a PEG_3_ linker. Both compounds can conveniently be radiolabeled and show comparable high hydrophilicity. However, protein binding is almost doubled for [^68^Ga]Ga-**T0G3** compared to that for [^68^Ga]Ga-**T3G3** after 120 min of incubation. Furthermore, a strong correlation between linker length and the affinity for the ASGR can be observed. The linker-free derivative **T0G3** possesses an approximately 5 times lower binding affinity, which might contribute to its poor *in vivo* performance. To put it in a nutshell, only [^68^Ga]Ga-**T3G3** exhibited comparable liver uptake to [^68^Ga]Ga-NODAGA-TriGalactan (29.7 ± 1.7% ID/g vs 32.3 ± 3.7% ID/g, 30 min p.i.). Nevertheless, these results are in line with literature data suggesting that sufficient spatial separation between the sugar residues is required to obtain an optimal binding geometry for the ASGR.^17^ In addition, it is also less surprising that the introduction of PEG linkers increases the hydrodynamic radius of a compound and hence, minimizes lipophilic interactions of the molecule with serum proteins.^31^

According to the literature, *N*-acetylgalactosamine possesses 10–20 times higher affinity for the ASGR than galactose.^32,33^ To test the influence of saccharide selection on the overall performance, trimer **T3N3** was synthesized. Despite the introduction of three GalNAc moieties, *in vitro* data suggest that [^68^Ga]Ga-**T3N3** possesses hydrophilicity and stability comparable to those of [^68^Ga]Ga-**T3G3**. Solely protein binding was found to be significantly higher. However, the *in vitro* parameters alone did not sufficiently reveal the full potential of the compound. To be precise, the IC_50_ value found for [^nat^Ga]Ga-**T3N3** was actually comparable to that of [^nat^Ga]Ga-**T3G3**. One possible explanation might be the fact that the IC_50_ assay does not fully depict the receptor’s true morphology. The ASGR is known to be a trimer comprising two H1 and one H2 subunit. However, the assay here is solely based on the immobilized H1 subunit. It might be possible that this leads to modified binding capabilities, resulting in altered IC_50_ values. Nevertheless, **T3N3** and **T3G3** both exhibit low nanomolar affinity for ASGR, while **T0G3** shows, as expected, an elevated IC_50_ value. The specificity of the assay was furthermore demonstrated by the fact that no affinity for trimer **T3U3** could be detected. The latter compound features glucose on a PEG_3_ linker and serves as a global negative control as glucose is known to not have any affinity for the ASGR.^32,33^

The trimers’ imaging properties were better characterized by the biodistribution data. Here, [^68^Ga]Ga-**T3N3** showed the highest liver uptake. In addition, maximum values were already reached 10 min p.i. while blood activity concentration was already very low at that time point. Compared to the other tracers in this study, [^68^Ga]Ga-**T3N3** showed the least accumulation in nontarget tissue and therefore resulted in excellent liver-to-organ ratios. Even kidney uptake is at a global minimum, indicating that the majority of the compound gets indeed taken up during its first passage through the liver. This rapid and massive liver uptake confirms the finding that the replacement of galactose by *N*-acetylgalactosamine can have a positive impact on the binding potential toward the ASGR.

To demonstrate specificity for this new class of radiopharmaceuticals, we synthesized the negative control compound [^68^Ga]Ga-**T3U3** bearing glucose instead of galactose or GalNAc. Indeed, [^68^Ga]Ga-**T3U3** showed almost no activity accumulation in the liver, while rapid renal elimination was maintained. As a result, an overall low activity concentration in all organs was found representing solely the amount of unspecific binding of this tracer class in the body. This finding visualizes the fact that glycoside selection is indeed a decisive factor in designing highly efficient ASGR-targeting compounds.

Another standard experiment to get information on the target specificity of a newly designed compound is based on the co-injection of a known endogenous ligand in excess. There is a variety of suitable compounds described for the ASGR.^34^ However, the commercial availability of such ligands in sufficient amounts is limited. Thus, we decided to include some blocking experiments using excess monosaccharides d-galactose and *N*-acetylgalactosamine. Indeed, a significant reduction in liver uptake was observed upon co-injection of the blocking agent for all three derivatives, with the highest effect seen for [^68^Ga]Ga-**T3N3**. It also appears to the critical viewer that especially for [^68^Ga]Ga-**T3N3** uptake in the intestine, stomach, pancreas, and partial muscle is reduced as well with the most prominent effect found in the intestine. One possible explanation here might be the fact that some part of the tracer or tracer fragments gets biliary excreted upon internalization into the hepatocyte. Thus, reducing the uptake in the liver might also reduce the amount of compound flowing through the bile duct into the intestine. Anyway, we do not assume that this slight biliary elimination influences the diagnostic interpretation. In the case of [^99m^Tc]Tc-GSA, we even found higher and more increasing activity concentrations in the intestine.^22^ However, this issue has not been considered a problem for the predictive outcome in described diagnostic approaches.^35^ For the significant activity reduction found for [^68^Ga]Ga-**T3N3** in the stomach, pancreas, and muscle, which is not that pronounced for [^68^Ga]Ga-**T3G3** and [^68^Ga]Ga-**T0G3**, no satisfactory explanation has been found yet. It is not assumed that it reflects target receptor expression, because in this case, the same effect should be present for all three compounds. Moreover, it has to be pointed out that the absolute uptake in these organs ranges between 0.7 and 1.5% ID/g 30 min p.i. and thus, is at least 50 times lower compared to the liver.

## Conclusions

TRIS-based Gal/GalNAc trimers have emerged as useful tools for addressing the ASGR for either imaging or drug delivery. However, their synthesis can be tedious and requires several HPLC purification steps. The TRAP chelator has been reported as an efficient tool for the generation of multimeric PET tracers, addressing a variety of different targets. To the best of our knowledge, no ASGR tracer featuring this chelator has been reported so far. Therefore, within this study, a small set of TRAP-based glycoside trimers has been synthesized, and their suitability as imaging agents for this receptor has been evaluated. Of all compounds tested [^68^Ga]Ga-**T3N3** showed the highest liver uptake, while the least off-target binding was observed resulting in a superior pharmacokinetic profile. Imaging studies in C57BL/6J mice confirmed a rapid elimination from the circulation and allowed for a clear delineation of the functional liver tissue. Therefore, we can conclude that TRAP-based carbohydrate trimers extend the palette of small-molecule-based ASGR imaging agents. [^68^Ga]Ga-**T3N3** revealed even better imaging properties than our first-generation compound [^68^Ga]Ga-NODAGA-TriGalactan. Thus, [^68^Ga]Ga-**T3N3** is a new radiopharmaceutical for the determination of functional liver mass with imaging properties comparable to those of the gold standard [^99m^Tc]Tc-GSA.

## Experimental Section

All reagents were obtained from VWR International GmbH (Vienna, Austria) or Merck (Darmstadt, Germany) and used as supplied without further purification. Triazacyclonoane triphosphinate (TRAP) was obtained from Fosfinos (Prague, Czech Republic). 1-Azido-1-deoxy-β-d-galactopyranoside tetraacetate, 2-[2-(2-azidoethoxy)ethoxy]ethyl 2,3,4,6-tetra-*O*-acetyl-d-galactopyranoside, 2-[2-(2-azidoethoxy)ethoxy]ethyl 2-acetamido-2-deoxy-β-d-galactopyranoside, and 2-[2-(2-azidoethoxy)ethoxy]ethyl β-d-glucopyranoside were obtained from Merck. The ^68^Ge/^68^Ga generator was purchased from Eckert & Ziegler (Berlin, Germany) with a nominal activity of 1.85 GBq and was eluted with 0.1 N HCl (GMP grade, Rotem Industries Ltd., Mishem Yamin D. N. Arava, Israel). Human recombinant ASGR1 (#4394-AS) was obtained from R&D Systems (Minneapolis). Human α_1_-glycoprotein was commercially available at Merck. Iodine-125 was supplied from PerkinElmer (Waltham, MA) at an activity of 37 MBq. Radio thin-layer chromatography (TLC) was performed with iTLC-SG stripes (Agilent Technologies, Santa Clara, CA) using either 0.1 M Na-citrate or 1 M Na-acetate/MeOH (1:1, v/v) as the eluents. For readout of the stripes, a ScanRAM radio-TLC scanner (LabLogic, Broomhill, Sheffield, U.K.) was used. Radioactivity of the samples was quantified in a 2480 Wizard^2^ 3″ γ-counter (PerkinElmer, Waltham, MA). Analytical HPLC was performed on an UltiMate 3000 chromatography system (Thermo Fisher, Waltham, MA) equipped with a WPS-3000SL autosampler, quarternary LPG-3400SD pump, and a variable wavelength detector at a flow rate of 1 mL/min. The column in use was a Dr. Maisch (Ammerbuch Entringen, Germany) ReproSil Pur C_18_ AQ 150 × 4.6 mm, 5 μm, 120 Å. For semipreparative HPLC applications, an UltiMate 3000 chromatography system (Thermo Fisher) equipped with a binary RS Pump and a variable wavelength detector was used at a flow rate of 8 mL/min. The column in use was a Dr. Maisch ReproSil Pur C_18_ AQ 250 mm × 20 mm, 5 μm, 120 Å. All devices were controlled with Chromeleon 7 software. Degassed water (solvent A) and acetonitrile (solvent B) both containing 0.1% (v/v) TFA served as eluents. Mass spectra were recorded on a Bruker (Billerica, MA) Microflex Benchtop MALDI-TOF MS instrument in positive or negative ionization mode. Therefore, samples were mixed 1:1 (v/v) with a saturated solution of α-cyano-4-hydroxycinnamic acid containing 0.1% (v/v) TFA, placed on a steel target matrix, and dried under a stream of argon. Additional mass spectra were recorded on a Thermo Scientific LTQ Velos ESI-MS device with water (solvent A) and acetonitrile (solvent B) containing 0.1% formic acid as eluents. NMR spectra were recorded on an Avance 4 Neo spectrometer (Bruker) at 700 MHz (^1^H) and 176 MHz (^13^C) in D_2_O (Eurisotop, Saarbrücken, Germany). Chemical shifts are given in parts per million (ppm) and are calibrated on the residual solvent signal. Coupling constants (*J*) are reported in hertz (Hz), and multiplicities are denoted as follows: s—singlet, d—doublet, t—triplet, q—quartet, m—multiplet.

Experimental procedures for stability studies in serum and PBS, log *D* determination, and protein binding have been described elsewhere.^36^ All compounds are ≥94% pure by HPLC analysis.

### TRAP(Alkyne)_3_

TRAP-Pr (200 mg, 345 μmol, 1.0 equiv) was dissolved in 1 mL of DMSO, and 724 μL of DIPEA (4.16 mmol, 12.0 equiv) was added with stirring. Once the solution had cleared up, propargylamine hydrochloride (158 mg, 1.73 mmol, 5.0 equiv) and HATU (1186 mg, 3.12 mmol, 9.0 equiv) were added as solids. After 1 h at room temperature, all volatiles were removed *in vacuo*, and the residue was transferred to a 15 mL centrifuge tube, followed by the addition of 1.5 mL of Millipore water and 25 μL of TFA. Precipitates were centrifuged off (3000 rpm, 10 min), and the supernatant was directly subjected to purification via semipreparative HPLC (5–15% B in 30 min). Lyophylization of the product fractions yielded 232 mg (336 μmol, 97%) of a colorless oil. Calculated monoisotopic mass (C_27_H_45_N_6_O_9_P_3_): 690.60 Da; **found** (*m*/*z*) = 691.3 [M + H]^+^, 713.2 [M + Na]^+^. ^**1**^**H NMR** (700 MHz, D_2_O): δ (ppm) = 3.97 (d, ^3^*J* = 2.7 Hz, 5H, f), 3.45 (s, 12H, a), 3.36 (d, ^3^*J* = 5.6 Hz, 7H, b), 2.61 (t, ^3^*J* = 2.6 Hz, 1H, f), 2.62–2.52 (m, 6H, c), 2.04–1.99 (m, 6H, d). ^**13**^**C NMR** (176 MHz, D_2_O): δ (ppm) = 175.3/175.2 (c-e), 163.8/163.6/163.3 (c-h), 119.9/118.0/116.0 (c-g), 80.3 (c-f), 72.5 (c-f), 55.2/54.6 (c-b), 52.2 (c-a), 28.6 (c-d), 26.5/25.9 (c-c).

### General Method for the Preparation of TRAP-Based Glycoside Trimers

A detailed synthesis description for each compound can be found in the Supporting Information. In general, TRAP(Alkyne)_3_ (3–10 mg, 1 equiv) was dissolved in 100 μL of Millipore water and mixed with a methanolic solution of the respective sugar azide (3.6 equiv). Next, solutions of copper acetate (1.2 equiv) and sodium ascorbate (40 equiv) in a minimum amount of water were added. This mixture was heated to 60 °C for 1 h. Removal of copper species was achieved by transchelation. Therefore, DTPA (30 equiv) was dissolved in 200 μL of Millipore water, and this solution was added to the reaction mixture. The pH was adjusted to 2.2 using concentrated HCl, and the reaction was allowed to progress for 1 h at 60 °C. LC-MS monitored the completion of the demetalation reaction. Purification of the trimers was achieved via semipreparative HPLC. Acetyl-protected trimers **T0G3** and **T3G3** were incubated overnight at room temperature in 3 mL of a mixture of triethylamine/methanol/water (1:6:2) (v/v/v). All volatiles were removed *in vacuo*, and the residue was subjected to a final purification via semipreparative HPLC.

After lyophilization, **T0G3** was obtained as a colorless solid (4.8 mg, 3.67 μmol, 25%). Calculated monoisotopic mass (C_45_H_78_N_15_O_24_P_3_): 1305.46 Da; **found** (*m*/*z*) = 926.2 [2(M + 2Na + K)]^+3^, 1305.8 [M + H]^+^, 1421.5 [M + H + 3K]^+^. ^**1**^**H NMR** (700 MHz, D_2_O): δ (ppm) = 8.19 (s, 3H, h), 5.68 (d, ^3^*J* = 9.3 Hz, 3H, i), 4.52 (s, 6H, f), 4.22 (t, ^3^*J* = 9.5 Hz, 3H, j), 4.10 (d, ^3^*J* = 3.3 Hz, 3H, l), 4.01 (t, ^3^*J* = 7 Hz, 3H, m), 3.89 (dd, ^3^*J* = 9.8/3.3 Hz, 3H, k), 3.80–3.79 (m, 6H, n), 3.49 (s, 12H, a), 3.28 (d, ^3^*J* = 5.8 Hz, 6H, b), 2.54–2.49 (m, 6H, c), 1.97–1.92 (m, 6H, d). ^**13**^**C NMR** (176 MHz, D_2_O): δ (ppm) = 175.8 (c-e), 145.7 (c-g), 123.5 (c-h), 88.7 (c-i), 78.9 (c-m), 73.6 (c-k), 70.4 (c-j), 69.3 (c-l), 61.5 (c-n), 55.3/54.6 (c-b), 52.1 (c-a), 35.3 (c-f), 28.9 (c-c), 27.2/26.6 (c-d).

After lyophilization, **T3G3** was obtained as a hygroscopic solid (2.0 mg, 1.2 μmol, 5%). Calculated monoisotopic mass (C_63_H_114_N_15_O_33_P_3_): 1701.69 Da; **found** (*m*/*z*) = 1748.1 [M + H + 2Na]^+^. ^**1**^**H NMR** (700 MHz, D_2_O): δ (ppm) = 7.98 (t, ^3^*J* = 9.1 Hz, 3H, h), 4.04 (dt, ^3^*J* = 11.8/4.4 Hz, 3H, q), 3.97 (t, ^3^*J* = 5.0 Hz, 6H, f), 3.93 (d, ^3^*J* = 3.7 Hz, 3H, o), 3.81–3.75 (m, 15H, a/q), 3.70–3.65 (m, 38H, i/k/l/n/p), 3.54 (t, ^3^*J* = 7.0 Hz, 3H, m), 3.21 (s, 6H, j), 2.84 (d, ^3^*J* = 11.6 Hz, 6H, b), 2.60–2.54 (m, 6H, c), 1.98–1.94 (m, 6H, d). ^**13**^**C NMR** (176 MHz, D_2_O): δ (ppm) = 163.8 (c-e), 125.1 (c-h), 117.8 (c-g), 103.5 (c-l), 75.9 (c-p), 73.4 (c-n), 71.5 (c-m), 70.3 (c-a/c-i), 69.4 (c-o/c-q), 69.3 (c-j/c-k), 61.6 (c-q), 53.4 (c-b), 50.7 (c-i), 35.1 (c-f), 30.6 (c-c), 27.8 (c-d).

After lyophilization, **T3N3** was obtained as a colorless solid (880 μg, 482 nmol, 10%). Calculated monoisotopic mass (C_69_H_123_N_18_O_33_P_3_): 1824.77 g/mol; **found** (*m*/*z*) = 1823.8 [M – H]^−^, 1885.5 [M – H + Na + K]^−^. ^**1**^**H NMR** (700 MHz, D_2_O): δ (ppm) = 8.03 (s, 3H, h), 4.63 (t, ^3^*J* = 7 Hz, 5H, j), 4.49 (s, 5H, f), 4.47 (d, ^3^*J* = 8.5 Hz, 3H, l), 3.97–3.94 (m, 9H, i/p), 3.92 (d, ^3^*J* = 3 Hz, 3H, o), 3.89 (dd, ^3^*J* = 10.8/8.5 Hz, 3H, m), 3.81–3.76 (m, 6H, k), 3.74–3.69 (m, 7H, n/k), 3.67–3.56 (m, 22H, q/k), 3.43 (s, 12H, a), 3.37 (d, ^3^*J* = 5.5 Hz, 6H, b), 2.56–2.51 (m, 6H, c), 2.04–2.00 (m, 6H, d), 1.99 (s, 9H, s). ^**13**^**C NMR** (176 MHz, D_2_O): δ (ppm) = 175.4 (c-e), 164.0/163.7/163.5 (c-r), 144.6 (c-g), 125.4 (c-h), 102.3 (c-l), 75.8 (c-p), 71.8 (c-n), 70.5 (c-k), 70.3 (c-q), 69.6 (c-k), 69.3 (c-i), 68.5 (c-o), 61.7 (c-k), 55.1/54.5 (c-b), 53.1 (c-m), 52.2 (c-a), 51.2 (c-j), 35.0 (c-f), 28.5 (c-c), 26.4/25.8 (c-d), 22.9 (c-s).

After lyophilization, **T3U3** was obtained as a colorless solid (2.4 mg, 1.42 μmol, 13%). Calculated monoisotopic mass (C_63_H_114_N_15_O_33_P_3_.): 1701.69 Da; **found** (*m*/*z*) = 678.8 [2(M – 5H)]^5–^, 1190.8 [2(M – H + 2Na + K)]^3–^, 1703.2 [M + H]^−^. ^**1**^**H NMR** (700 MHz, D_2_O): δ (ppm) = 7.97 (s, 3H, h), 4.61 (t, ^3^*J* = 5.0 Hz, 5H, i), 4.47 (t, ^3^*J* = 4.0 Hz, 9H, f/l), 4.02 (dt, ^3^*J* = 11.4/4.2 Hz, 3H, k), 3.97 (t, ^3^*J* = 5.1 Hz, 7H, j), 3.92 (dd, 12.4/2.2 Hz, 3H, q), 3.79 (dt, ^3^*J* = 11.5/4.7 Hz, 3H, k), 3.72 (dd, ^3^*J* = 12.3/6.0 Hz, 3H, q), 3.67–3.65 (m, 18H, k), 3.51–3.44 (m, 18H, a/n/p), 3.39 (t, ^3^*J* = 9.4 Hz, 3H, o), 3.31–3.28 (m, 9H, b/m), 2.53–2.49 (m, 6H, c), 1.98–1.93 (m, 6H, d). ^**13**^**C NMR** (176 MHz, D_2_O): δ (ppm) = 175.6 (c-e), 145.2 (c-g), 125.0 (c-h), 102.9 (c-l), 76.6 (c-p), 76.4 (c-n), 73.8 (c-m), 70.3 (c-k), 70.2 (c-o), 69.4 (c-j/c-k), 61.4 (c-q), 55.3 (c-b), 52.1 (c-a), 50.7 (c-i), 35.3 (c-f), 28.8 (c-c), 27.2 (c-d).

### Radiolabeling

Labeling with ^68^Ga was carried out in 100 μL of a 5 M HEPES buffer (pH 5.8). For elution of the ^68^Ge/^68^Ga generator, a fractionated protocol was applied as reported before.^37^ Briefly, 550 μL (approximately 80–90 MBq) of ^68^Ga-eluate were added to 5 nmol (5 μL, 1 mM) of precursor in 100 μL of buffer. The mixture was incubated for 15 min at 95 °C under vigorous shaking (1300 rpm). Completion of the labeling reaction was monitored via radio-TLC and radio-HPLC using a GabiStar radio detector (Raytest, Straubenhardt, Germany). If required, the labeled compound was further purified via fixation on a Waters (Milford, MA) Sep Pak C_18_ Plus Light cartridge.

### ^nat^Ga-Complexation

Complexation with ^nat^Ga was performed in analogy to a previously published procedure.^38^ In brief, 200 μL (200 nmol, 1 mM) of an aqueous stock solution was mixed with 15 μL (300 nmol, 20 mM) of a ^nat^GaBr_3_ solution. Formation of the nonradioactive gallium complexes occurred instantly at room temperature.

#### ^nat^Ga-**T0G3**

RP-HPLC (1–10% B in 15 min): *t*_R_ = 9.7 (26%), 10.7 min (74%). Calculated monoisotopic mass (C_45_H_75_GaN_15_O_24_P_3_): 1371.6 Da; **found** (*m*/*z*) = 1395.1 [M + H + Na]^+^, 1412.9 [M + H + K]^+^.

#### ^nat^Ga-**T3G3**

RP-HPLC (5–15% B in 15 min): *t*_R_ = 12.0 (26%), 12.7 min (74%). Calculated monoisotopic mass (C_63_H_111_GaN_15_O_33_P_3_): 1767.6 Da; **found** (*m*/*z*) = 1791.3 [M + H + Na]^+^, 1807.3 [M + H + K]^+^.

#### ^nat^Ga-**T3N3**

RP-HPLC (5–15% B in 15 min): *t*_R_ = 14.5 (25%), 14.9 min (75%). Calculated monoisotopic mass (C_69_H_120_GaN_18_O_33_P_3_): 1890.7 Da; **found** (*m*/*z*) = 947.1 [M + 2H]^2+^, 631.6 [M + 3H]^3+^.

#### ^nat^Ga-**T3U3**

RP-HPLC (5–15% B in 15 min): *t*_R_ = 14.1 min (31%), 14.7 min (69%). Calculated monoisotopic mass (C_63_H_111_GaN_15_O_33_P_3_): 1767.6 Da; **found** (*m*/*z*) = 1198.5 [2M + 3Na]^3+^, 885.7 [M + 2H]^2+^, 590.6 [M + 3H]^3+^.

### Solid-Phase Binding Assay

Affinity studies (IC_50_) were performed in triplicates using an isolated receptor-based assay.^32^ A more detailed description of coating, blocking, and washing steps can be found in the Supporting Information. In brief, human recombinant ASGR1 was immobilized overnight in 24 wells of a flat-bottom 96-well plate and dilutions of nonradioactive gallium complexes in PBS (10^–4^–10^–10^) as well as ^125^I-ASOR (50 nM, ∼150 000 cpm/well) were added. After 1 h of incubation at room temperature, supernatants were removed and the wells were washed with TRIS-buffered saline (TBS). Release of surface-bound activity was achieved by incubating the wells for 10 min with hot (60 °C) 1 M NaOH. The wells were washed twice with 1 M NaOH and lysates, and wash fractions were combined. The activity content of the lysates was determined in a γ-counter (PerkinElmer, Waltham, MA). Values were plotted, and fitting of the sigmoidal binding curve was done using Excel’s solver plugin. IC_50_ values were calculated from at least three technical replicates.

### Biodistribution Data

All animal experiments were conducted in accordance with the Austrian animal experiment law (BGBl. I Nr. 114/2012) and according to the institution’s animal welfare standards as approved by the Austrian Federal Ministry of Education, Science and Research (BMBWF, 2022-0.311.708). For biodistribution studies, 6-week-old female BALB/c mice (*n* = 3, Charles Rivers Laboratories, Sulzfeld, Germany) were injected with 0.1 nmol (100 μL, approximately 1 MBq) of ^68^Ga-labeled compound via the lateral tail vein and sacrificed by cervical dislocation 10, 30, or 60 min post injection (p.i.). The mice were dissected, blood and organs were weighed, and the activity of the samples measured in a γ-counter. For blocking experiments, the mice were co-injected with either 27.7 μmol of d-galactose or *N*-acetylgalactosamine.

### Small Animal Imaging

All animal experiments were performed according to the German Animal Welfare Act and approved by the local authorities (R5/19 and R09/21 G). Male C57BL/6J mice were purchased from Janvier Lab (Saint-Berthevin Cedex, France) at an age of 6 weeks. The mice were kept under 1.5% isoflurane in oxygen during the measurements (*n* = 3). Approximately 1 MBq of the radiotracer was injected intravenously per mouse, and 1 h dynamic PET was performed with an Inveon microPET system (Inveon D-PET, Siemens, Knoxville, TN). The mice were then transferred to a 7 T BioSpec 70/30 USR (Bruker Biospin MRI GmbH, Ettlingen, Germany) and a 15 min MR anatomical scan using T_2_-weighted three-dimensional turbo RARE sequence was acquired. Inveon Acquisition Workplace was used to reconstruct the acquired images using ordered subset expectation maximization 3D (OSEM 3D) and framing the dynamics. The reconstructed image analysis was performed by using the Inveon Research Workplace. After co-registering acquired MR images with PET images, regions of interest (ROIs) were drawn on liver and heart, and time activity curves (TACs) were quantified.

### Statistical Analysis

Statistical significance of experimental data was calculated in SPSS using Student’s *t-*test for unpaired samples.

## Supporting Information Available

Synthesis of TRAP(Alkyne)_3_, characterization with RP-HPLC, MS, and NMR (Figures S1–S6); synthesis of T0G3, characterization with RP-HPLC, MS, and NMR (Figures S7–S12); synthesis of T3G3 characterization with RP-HPLC, MS, and NMR (Figures S13–S18); synthesis of T3N3, characterization with RP-HPLC, MS, and NMR (Figures S19–S24); synthesis of T3U3, characterization with RP-HPLC, MS, and NMR (Figures S25–S30); detailed description of the solid-phase binding assay using [^125^I]-asialoorosomucoid and human recombinant ASGR1 including coating, blocking and washing steps; and analytical HPLC traces of ^nat^Ga-T0G3 and equilibrium studies (Figure S31) (PDF)
Molecular formula strings (CSV)

## Abbreviations Used

% ID/g: percent injected dose per gramm
ASGR: asialoglycoprotein receptor
ASOR: asialoorosomucoid
D_2_O: deuterated water
DIPEA: diisopropylethylamine
eq: equivalents
Gal: galactose
GalNAc: *N*-acetylgalactosamine
GSA: galactosyl human serum albumin
HATU: 1-[Bis(dimethylamin)methylen]-1H-1,2,3-triazol[4,5-*b*]pyridinium 3-oxid hexafluorophosphate
HCl: hydrochloric acid
log *D*: *n*-octanol/PBS partition coefficient
MeCN: acetonitrile
MeOH: methanol
NAFLD: nonalcoholic fatty liver disease
NASH: nonalcoholic staeto hepatitis
NaOH: sodium hydroxide
NEt_3_: trimethylamine
NOTA: 2,2′,2″-(1,4,7-triazacyclononane-1,4,7-triyl)triacetic acid
p.i.: post injection
PSMA: prostate specific membrane antigen
RCP: radiochemical purity
RCY: radiochemical yield
ROI: region of interest
TAC: time activity curve
TBS: Tris-buffered saline
TRAP: 3,3′,3″-(((1,4,7-triazonane-1,4,7-triyl)tris(methylene))tris(hydroxyphosphoryl))tripropanoic acid
