# Supplementary material for: 68Ga-Labeled TRAP-Based Glycoside Trimers for Imaging of the Functional Liver Reserve
Source: J Med Chem. 2024 Oct 16;67(21):19668–77. doi: 10.1021/acs.jmedchem.4c02006 (PMC11571218; doi:10.1021/acs.jmedchem.4c02006)
Supplement: Supplementary file 1 — jm4c02006_si_001.pdf [file jm4c02006_si_001.pdf]

Supporting Information

^{68}Ga-labeled TRAP-based Glycoside Trimers for Imaging of the Functional Liver Reserve

Maximilian A. Zierke[1], Christine Rangger[1], Kimia Samadikhah[2], Christoph Kreutz[3], Andreas M. Schmid[2], Roland Haubner[1,*]

[1]Department of Nuclear Medicine, Medical University Innsbruck, Anichstr. 35, 6020 Innsbruck, Austria

[2]Werner Siemens Imaging Center, Department of Preclinical Imaging and Radiopharmacy, Eberhard Karls University Tübingen, Röntgenweg 13, 73076 Tübingen, Germany

[3]Institute of Organic Chemistry and Center for Molecular Biosciences (CMBI), University of Innsbruck, Innrain 80-82, 6020 Innsbruck, Austria.

*to whom correspondence should be addressed

roland.haubner@i-med.ac.at

Table of content

Synthetic Procedures and Analytical Data 2
TRAP(Alkyne)$_3$ 2
T0G3 6
T3G3 10
T3N3 14
T3U3 18
Solid-Phase Binding Assay 22
Equilibrium studies on TRAP-Conformers 23
Literature 23

Synthetic Procedures and Analytical Data

TRAP(Alkyne)$_3$

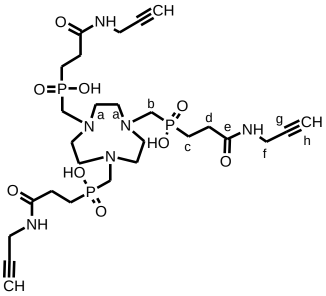

TRAP(Alkyne)$_3$

Formula Weight: 690.60 g/mol
Monoisotopic Mass: 690.25 Da
Molecular Formula: $C_{27}H_{45}N_6O_9P_3$

TRAP-Pr (200 mg, 345 µmol, 1.0 eq) was dissolved in 1 mL DMSO and 724 µL DIPEA (4.16 mmol, 12.0 eq) were added with stirring. Once the solution had cleared up, propargylamine hydrochloride (158 mg, 1.73 mmol, 5.0 eq) and HATU (1186 mg, 3.12 mmol, 9.0 eq) were added as solids. After 1 hour at room temperature, all volatiles were removed *in vacuo* and the residue was transferred to a 15 mL centrifuge tube, followed by the addition of 1.5 mL Millipore water and 25 µL TFA. Precipitates were centrifuged off (3000 RPM, 10 min) and the supernatant was directly subjected to purification via semipreparative HPLC (5-15 % B in 30 min). Lyophilization of the product fractions yielded 232 mg (336 µmol, 97 %) of a colourless oil.

Analytical HPLC (ReproSil Pur, 5-15 % B in 15 min, 1.0 mL/min) t_R = 11.0 min (12 % B).

ESI-MS (m/z) = 691.3 $[M+H]^+$, 713.2 $[M+Na]^+$.

^{1}H-NMR (700 MHz, D_2O): δ (ppm) = 3.97 (d, ^3J = 2.7 Hz, 5 H, f), 3.45 (s, 12 H, a), 3.36 (d, ^3J = 5.6 Hz, 7 H, b), 2.61 (t, 3J = 2.6 Hz, 1 H, f), 2.62-2.52 (m, 6 H, c), 2.04-1.99 (m, 6 H, d).

^{13}C-NMR (176 MHz, D_2O): δ (ppm) = 175.3/175.2 (c-e), 163.8/163.6/163.3 (c-h), 119.9/118.0/116.0 (c-g), 80.3 (c-f), 72.5 (c-f), 55.2/54.6 (c-b), 52.2 (c-a), 28.6 (c-d), 26.5/25.9 (c-c).

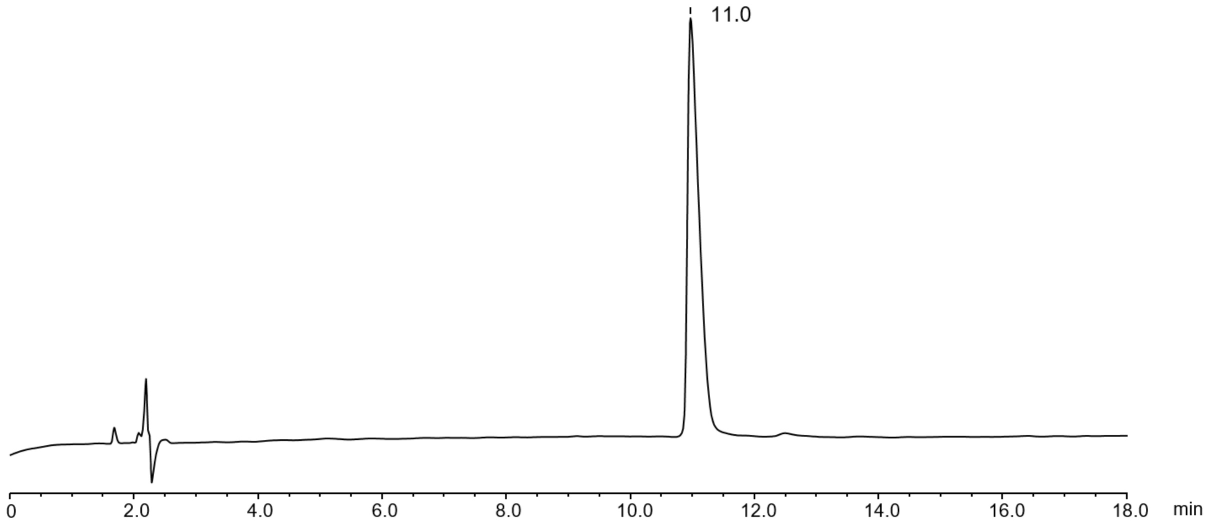

Fig. S1: RP-HPLC Chromatogram of TRAP(Alkyne)$_3$ at λ = 220 nm.

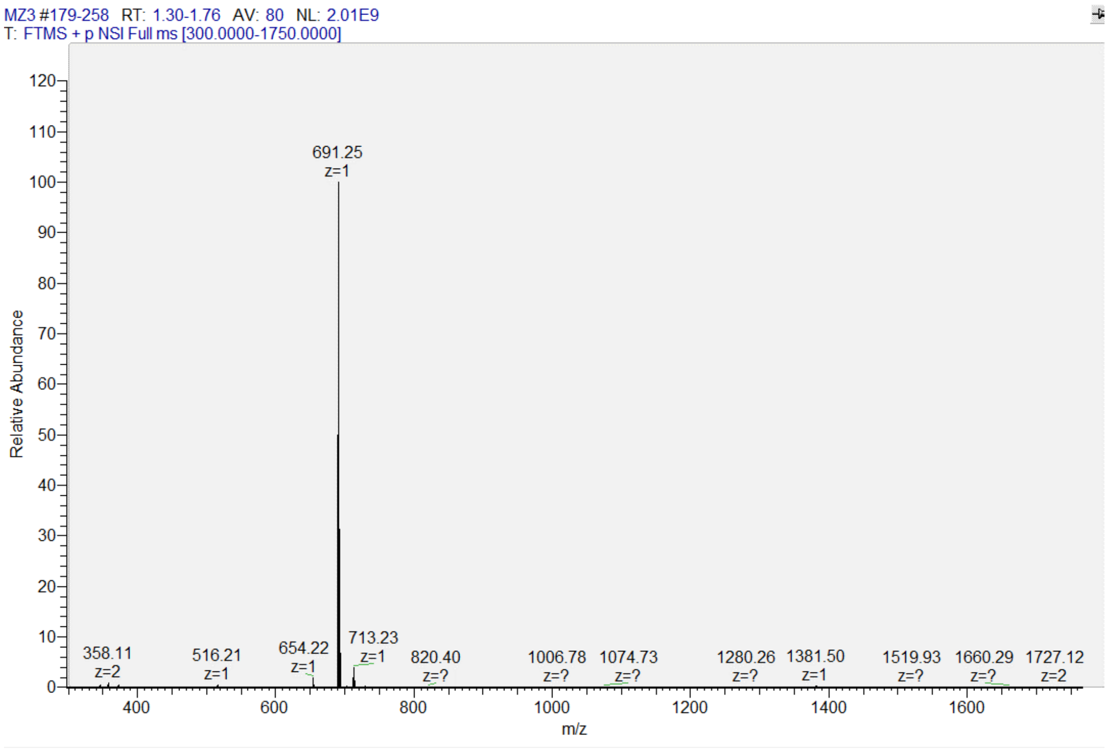

Fig. S2: Mass spectrum of TRAP(Alkyne)$_3$.

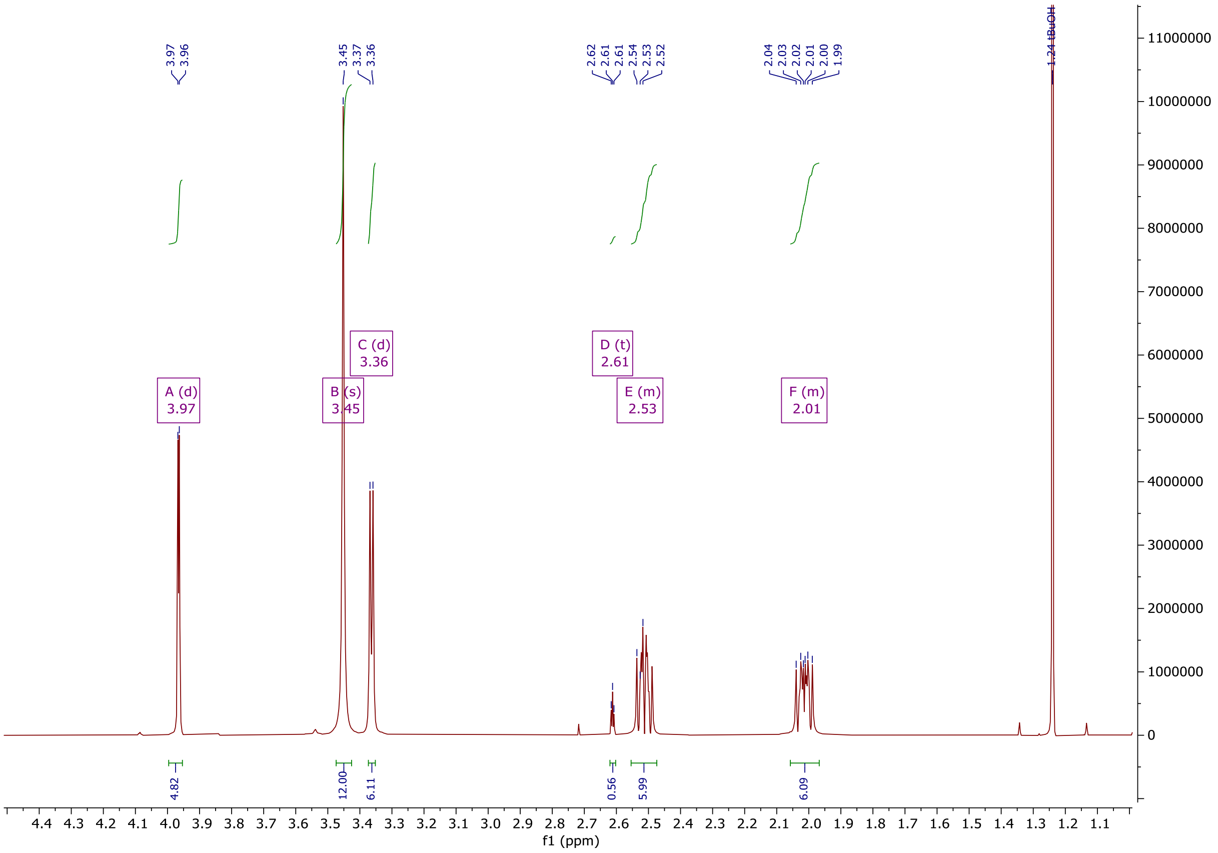

Fig. S3: ^{1}H-NMR spectrum of TRAP(Alkyne)$_3$.

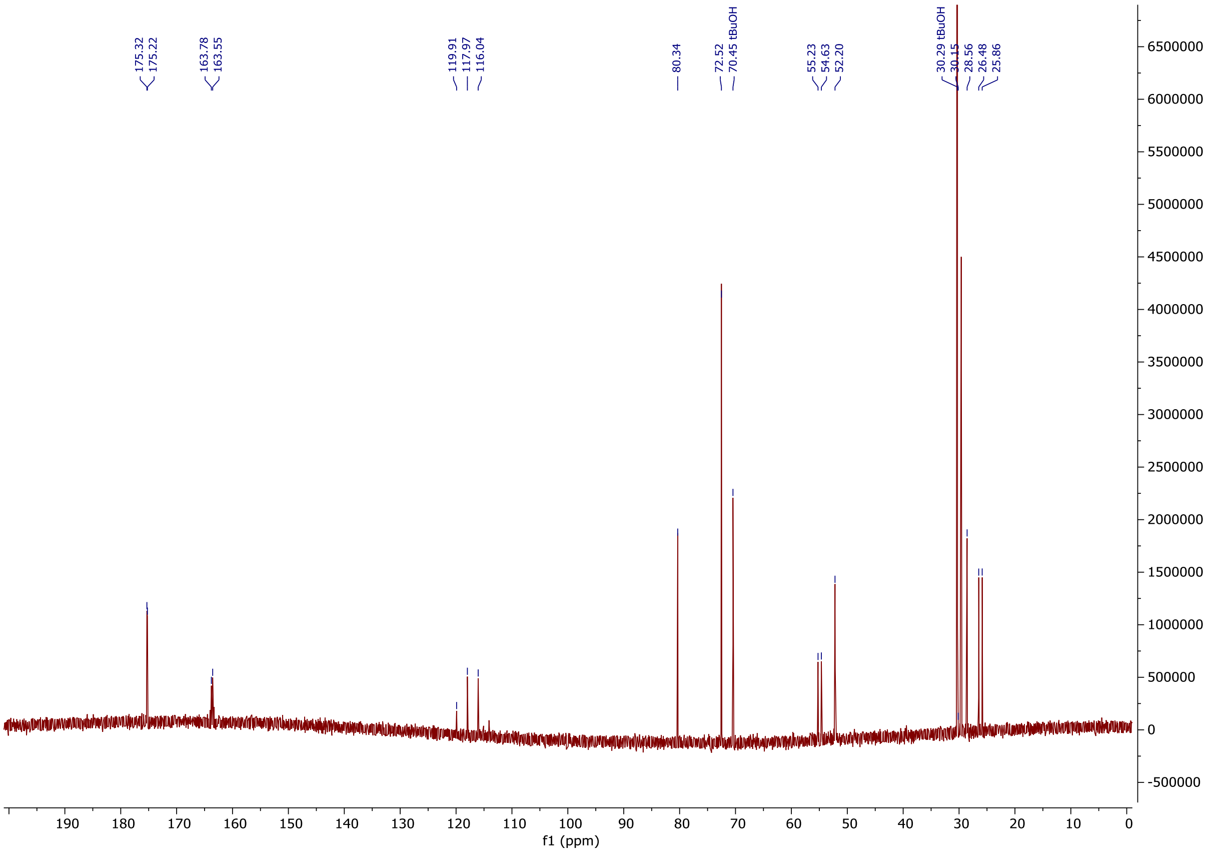

Fig. S4: ^{13}C{^{1}H}-NMR spectrum of TRAP(Alkyne)$_3$

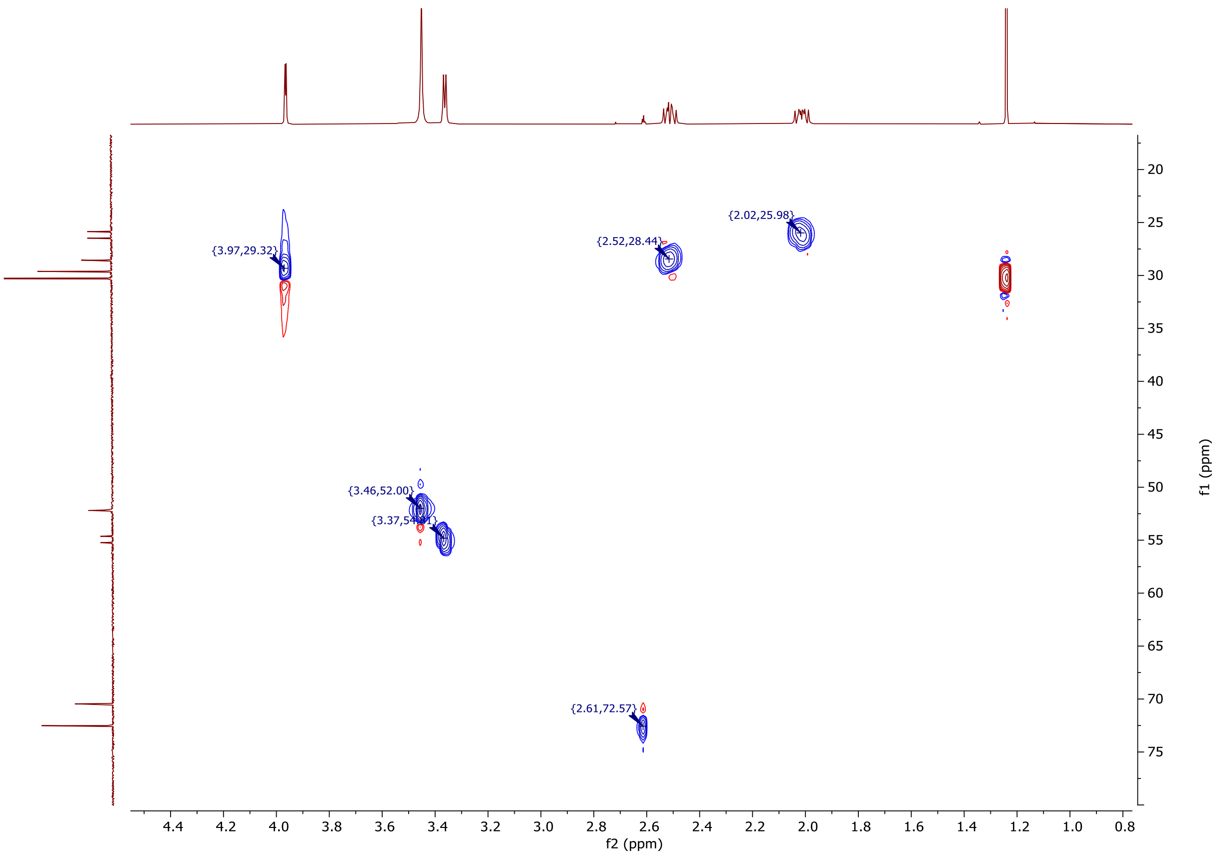

Fig. S5: HSQC-NMR spectrum for TRAP(Alkyne)$_3$ showing cross peaks between 0.8-4.4 ppm (^{1}H) and 20-80 ppm (^{13}C).

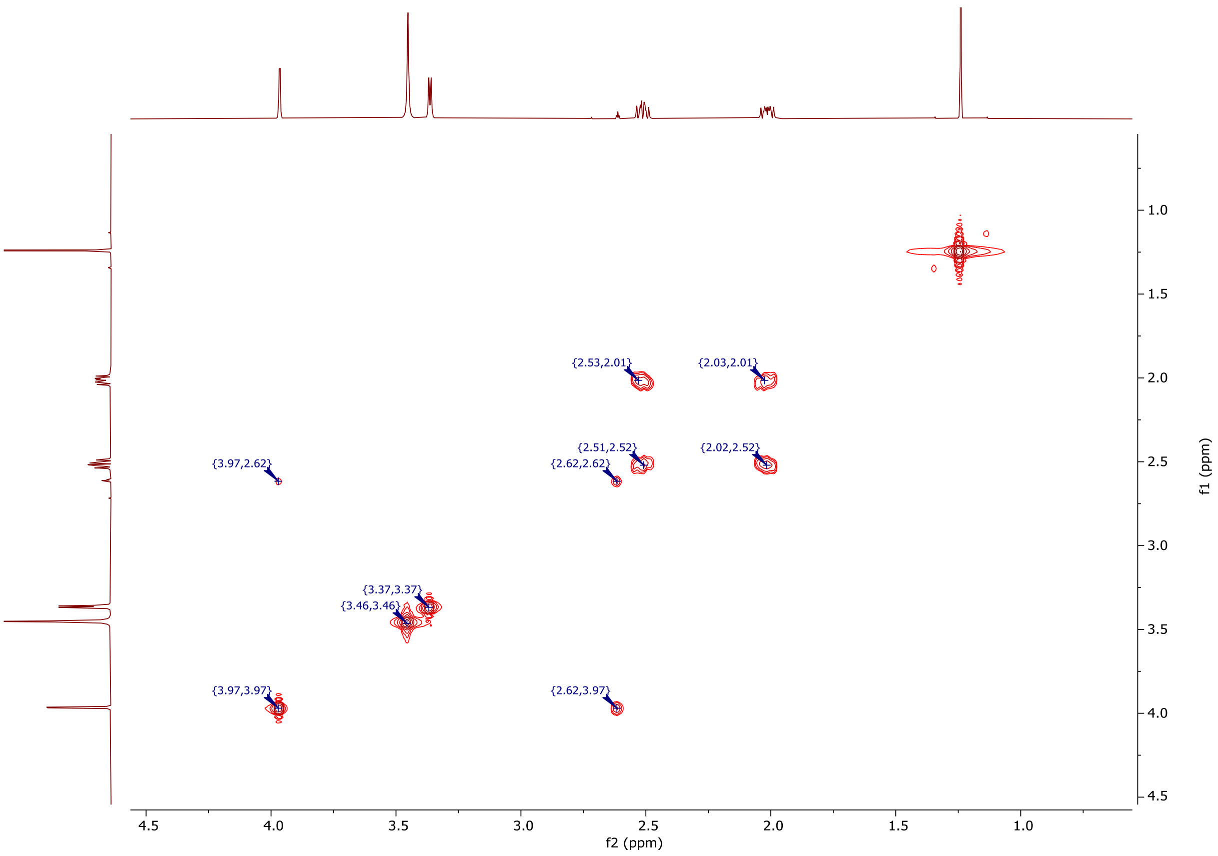

Fig. S6: {^{1}H, ^{1}H}-COSY spectrum of TRAP(Alkyne)$_3$ showing correlations between 1.0-4.5 ppm.

T0G3

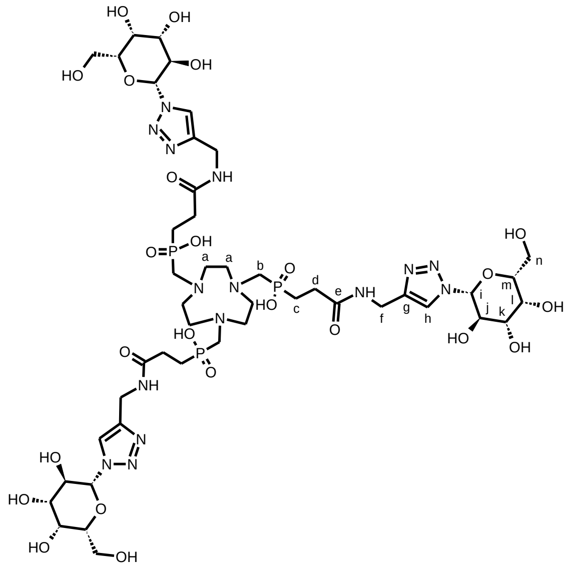

T0G3

Formula Weight: 1306.11 g/mol
Monoisotopic Mass: 1305.46 Da
Molecular Formula: $C_{45}H_{78}N_{15}O_{24}P_3$

TRAP(Alkyne)$_3$ (10.0 mg, 14.4 µmol, 1.0 eq) was dissolved in 120 µL of MeOH and mixed with a hot methanolic solution of 1-Azido-1-deoxy-β-D-galactopyranoside tetraacetate (16.7 mg, 44.6 µmol, 3.6 eq). Next, solutions of $Cu(OAc)_2$ (2.95 mg, 14.8 µmol, 1.2 eq) and sodium ascorbate (73.7 mg, 372 µmol, 30.0 eq) in a minimum amount of water were added. This mixture was incubated for 1 hour at 60 °C. For subsequent demetallation, a solution of DTPA (185 mg, 30.0 eq) in Millipore water was added and the pH was adjusted to 2.2 using concentrated HCl. After 1 hour at 60 °C the reaction mixture was directly injected into semipreparative HPLC (38 % B isocratic). For deacetylation the multimer was treated overnight with 3 mL of NEt_3/MeOH/H_2O (1:6:2). All volatiles were removed *in vacuo* and the residue was purified via semipreparative HPLC (1-10 % B in 30 min). Lyophilization yielded 4.8 mg (3.67 µmol, 25 %) of a colourless solid.

Analytical HPLC (ReproSil Pur, 1-10 % B in 15 min, 1.0 mL/min) t_R = 9.0 min (6 % B).

MALDI-MS (m/z) = 926.2 $[2(M+2Na+K)]^{+3}$, 1305.8 $[M+H]^+$, 1421.5 $[M+H+3K]^+$.

^{1}H-NMR (700 MHz, D_2O): δ (ppm) = 8.19 (s, 3 H, h), 5.68 (d, ^3J = 9.3 Hz, 3 H, i), 4.52 (s, 6 H, f), 4.22 (t, ^3J = 9.5 Hz, 3 H, j), 4.10 (d, ^3J = 3.3 Hz, 3 H, l), 4.01 (t, ^3J = 7 Hz, 3 H, m), 3.89 (dd, ^3J = 9.8/3.3 Hz, 3 H, k), 3.80-3.79 (m, 6 H, n), 3.49 (s, 12 H, a), 3.28 (d, ^3J = 5.8 Hz, 6 H, b), 2.54-2.49 (m, 6 H, c), 1.97-1.92 (m, 6 H, d).

^{13}C-NMR (176 MHz, D_2O): δ (ppm) = 175.8 (c-e), 145.7 (c-g), 123.5 (c-h), 88.7 (c-i), 78.9 (c-m), 73.6 (c-k), 70.4 (c-j), 69.3 (c-l), 61.5 (c-n), 55.3/54.6 (c-b), 52.1 (c-a), 35.3 (c-f), 28.9 (c-c), 27.2/26.6 (c-d).

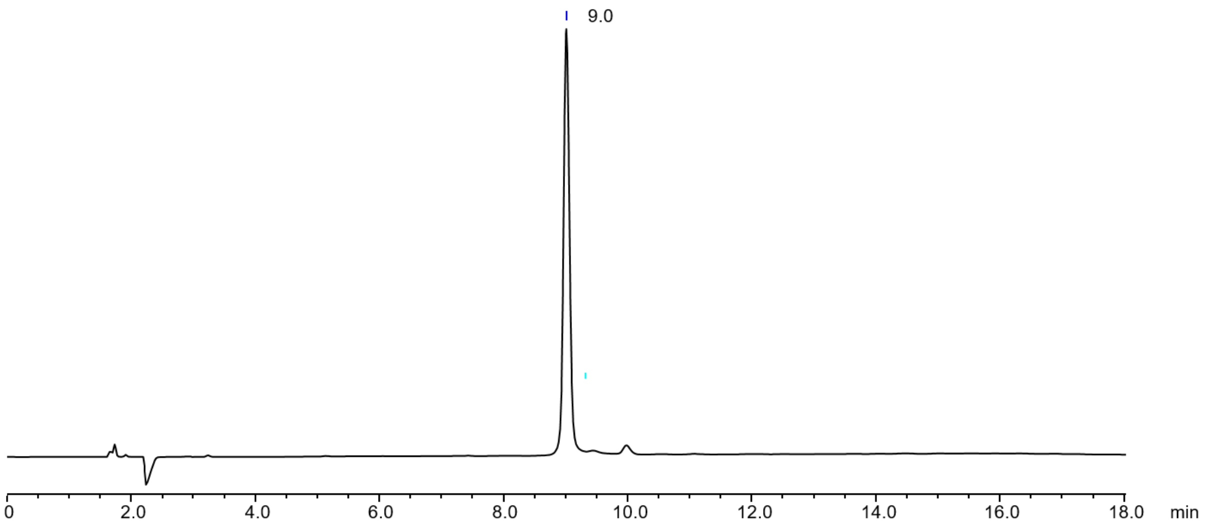

Fig. S7: RP-HPLC Chromatogram of T0G3 at λ = 220 nm.

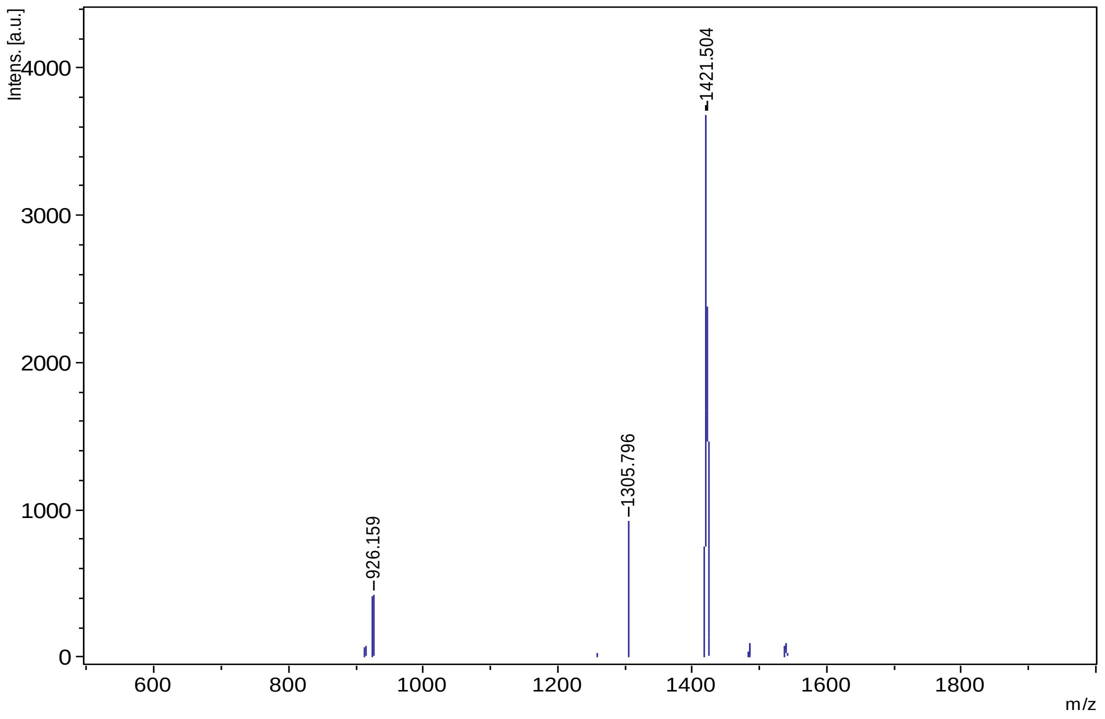

Fig. S8: Mass spectrum of T0G3.

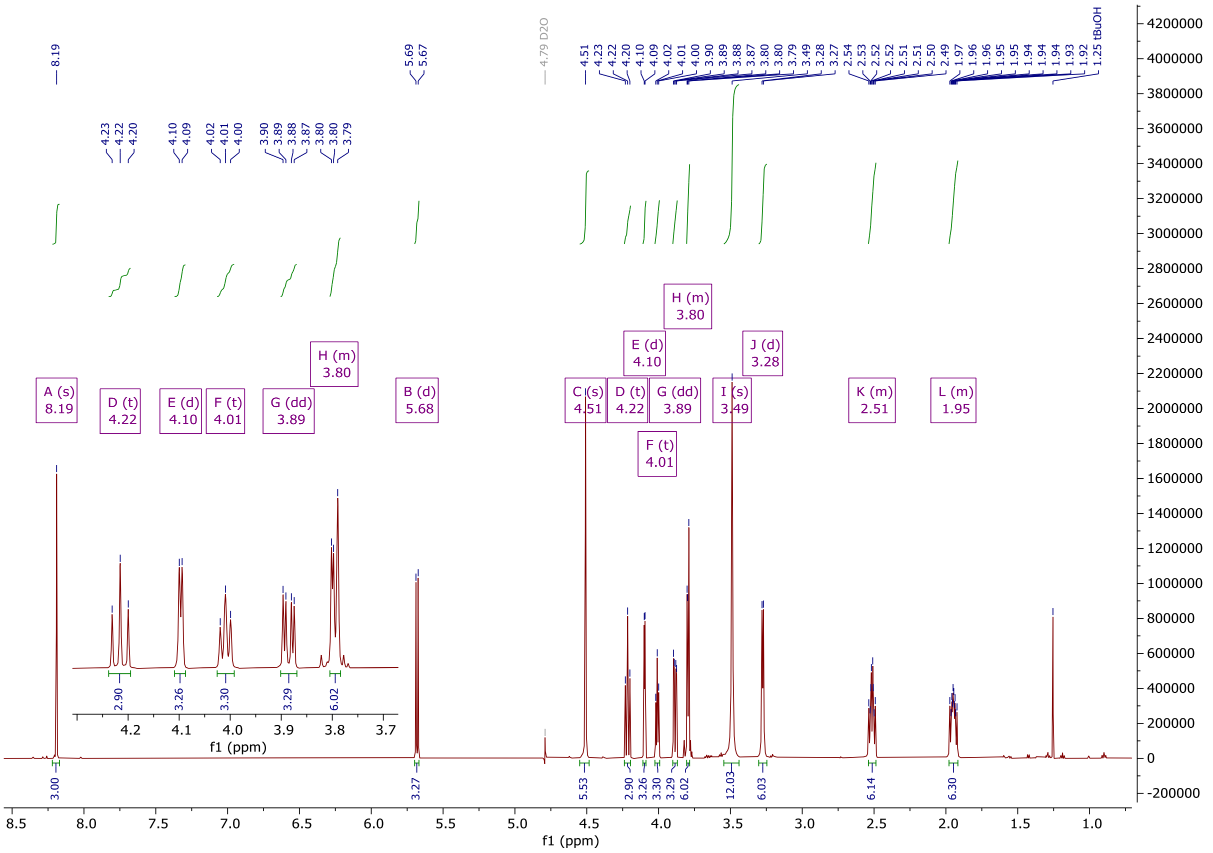

Fig. S9: ¹H-NMR spectrum of T0G3.

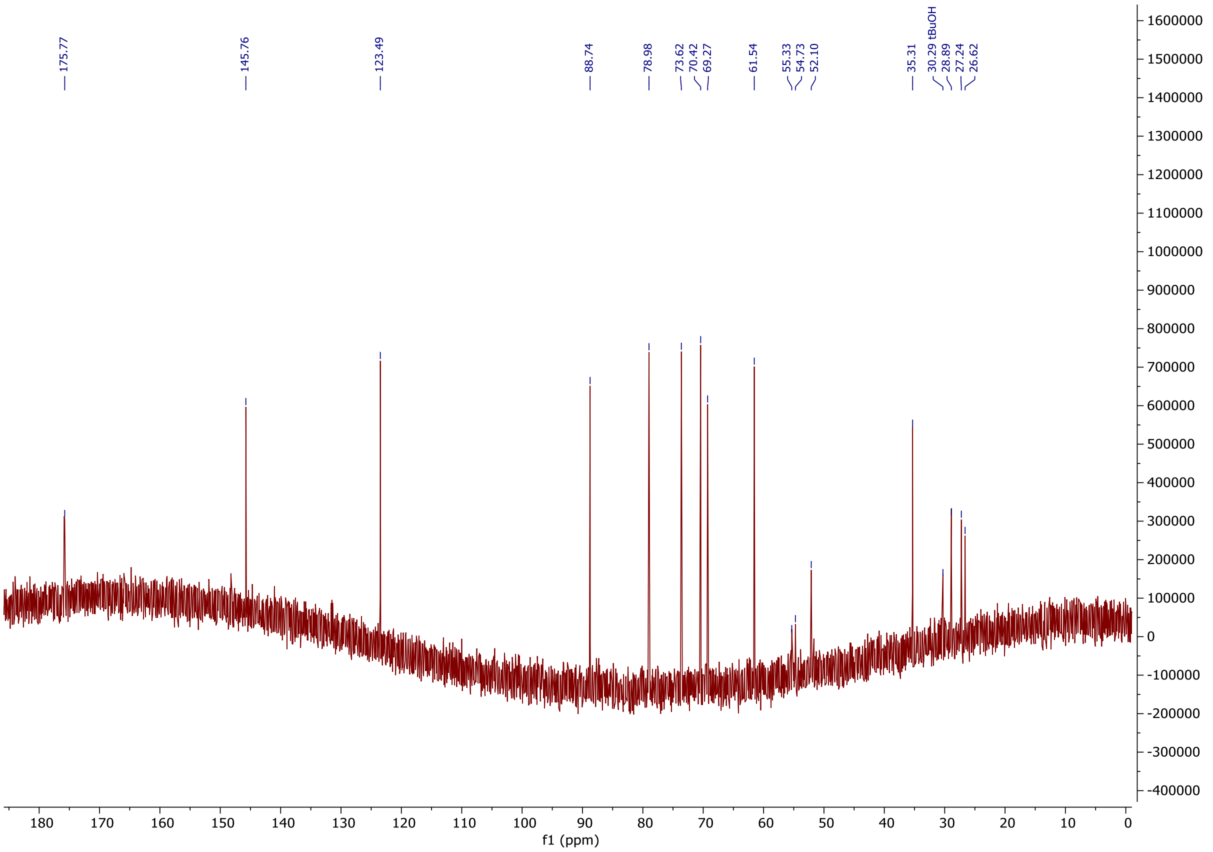

Fig. S10: $^{13}C\{^{1}H\}$-NMR spectrum of T0G3.

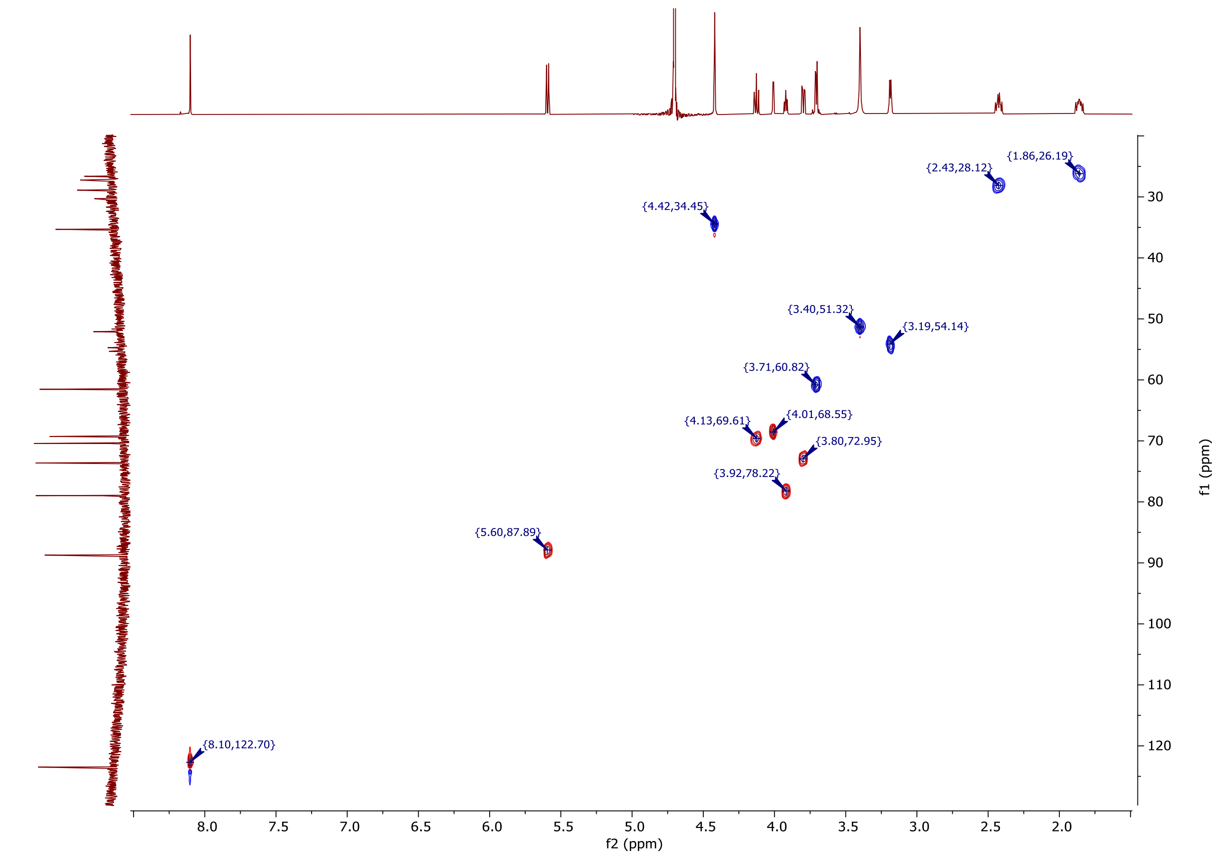

Fig. S11: HSQC-NMR spectrum of T0G3 showing cross peaks between 1.5-8.0 ppm (^{1}H) and 20-125 ppm (^{13}C).

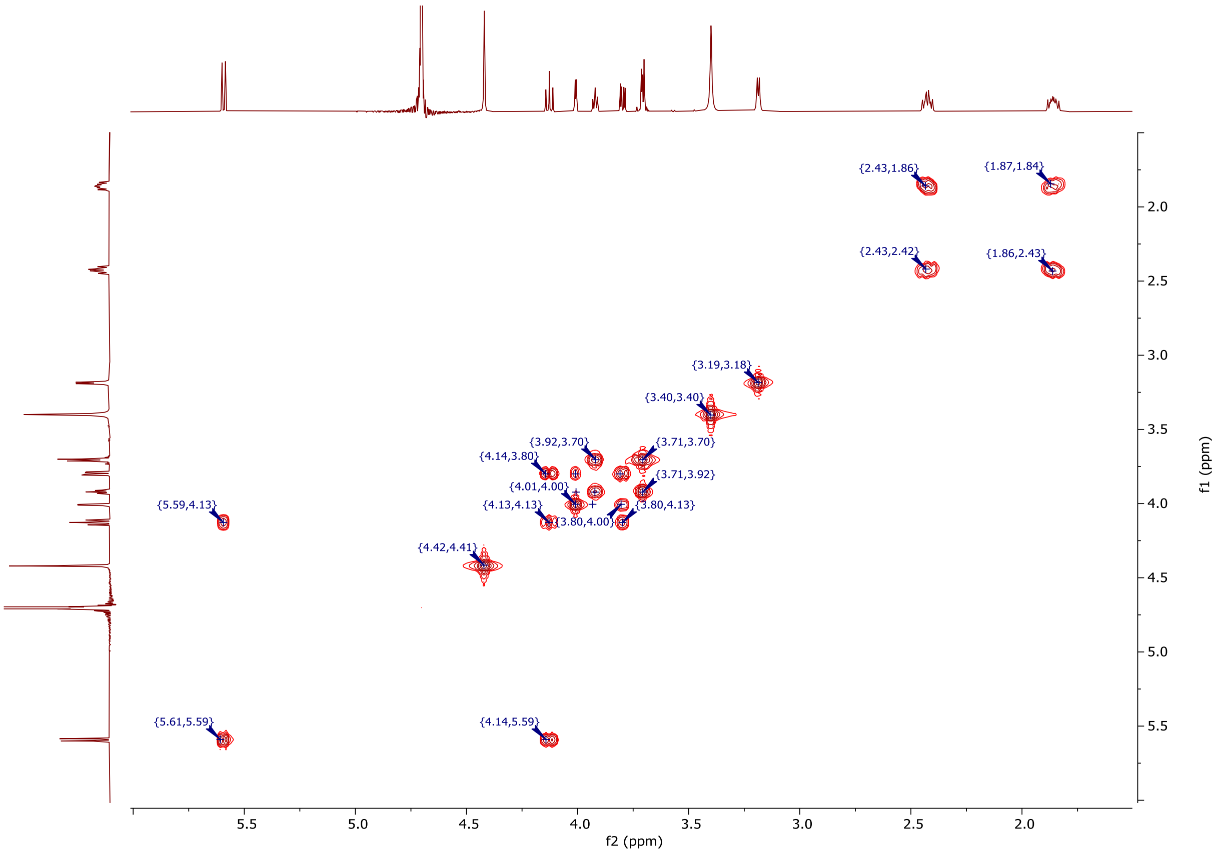

Fig. S12: {^{1}H, ^{1}H}-COSY spectrum of T0G3 showing correlations between 1.5-6.0 ppm.

T3G3

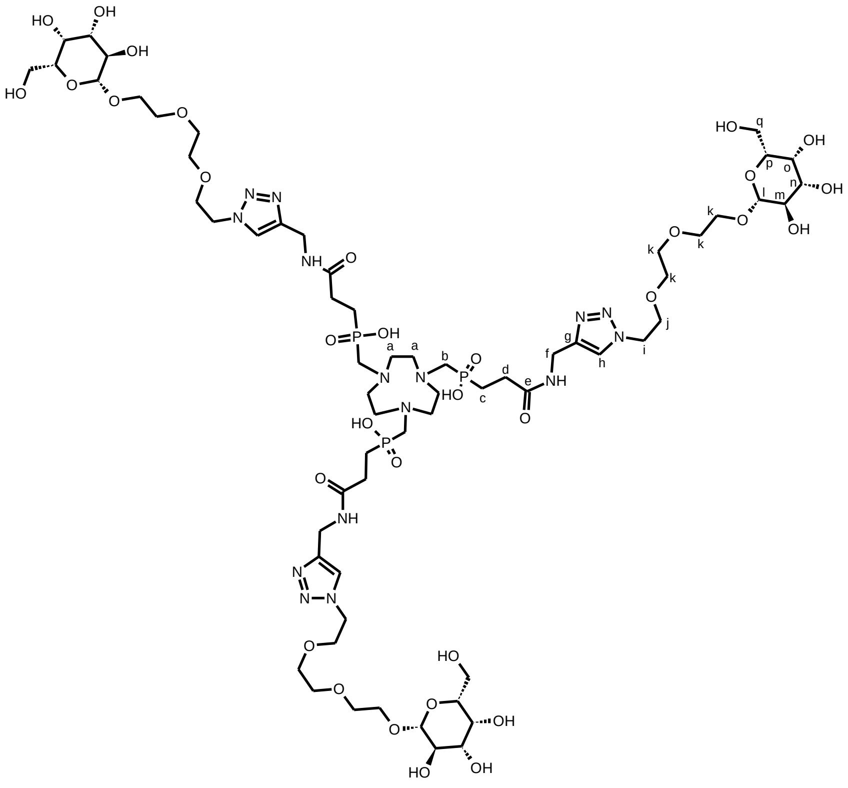

T3G3
Formula Weight: 1702.58 g/mol
Monoisotopic Mass: 1701.69 Da
Molecular Formula: $C_{63}H_{114}N_{15}O_{33}P_3$

TRAP(Alkyne)$_3$ (17.7 mg, 25.6 µmol, 1.0 eq) was dissolved in 250 µL of MeOH and mixed with 40.0 mg (79.1 µmol, 3.6 eq) of 2-[2-(2-Azidoethoxy)ethoxy]ethyl 2,3,4,6-Tetra-*O*-acetyl-D-galactopyranoside. Next, solutions of $Cu(OAc)_2$ (5.27 mg, 26.4 µmol, 1.2 eq) and sodium ascorbate (174 mg, 880 µmol, 40.0 eq) in a minimum amount of water were added. This mixture was incubated for 1 hour at 60 °C. For subsequent demetallation, a solution of DTPA (330 mg, 30.0 eq) in Millipore water was added and the pH was adjusted to 2.2 using concentrated HCl. After 1 hour at 60 °C the mixture was injected directly into semipreparative HPLC (38 % B isocratic). For deacetylation the multimer was treated overnight with 3 mL of NEt_3/MeOH/H_2O (1:6:2). All volatiles were removed *in vacuo* and the residue was purified with semipreparative HPLC (5-10 % B in 25 min). Lyophilization yielded 2.0 mg (1.2 µmol, 5 %) of a hygroscopic solid.

Analytical HPLC (ReproSil Pur, 5-15 % B in 15 min, 1.0 mL/min) t_R = 13.5 min (13 % B).

MALDI-MS (m/z) = 1748.1 $[M+H+2Na]^+$.

^{1}H-NMR (700 MHz, D_2O): δ (ppm) = 7.98 (t, ^3J = 9.1 Hz, 3 H, h), 4.04 (dt, ^3J = 11.8/4.4 Hz, 3 H, q), 3.97 (t, ^3J = 5.0 Hz, 6 H, f), 3.93 (d, ^3J = 3.7 Hz, 3 H, o), 3.81-3.75 (m, 15 H, a/q), 3.70-3.65 (m, 38 H, i/k/l/n/p), 3.54 (t, ^3J = 7.0 Hz, 3 H, m), 3.21 (s, 6 H, j), 2.84 (d, ^3J = 11.6 Hz, 6 H, b), 2.60-2.54 (m, 6 H, c), 1.98-1.94 (m, 6 H, d).

^{13}C-NMR (176 MHz, D_2O): δ (ppm) = 163.8 (c-e), 125.1 (c-h), 117.8 (c-g), 103.5 (c-l), 75.9 (c-p), 73.4 (c-n), 71.5 (c-m), 70.3 (c-a/c-i), 69.4 (c-o/c-q), 69.3 (c-j/c-k), 61.6 (c-q), 53.4 (c-b), 50.7 (c-i), 35.1 (c-f), 30.6 (c-c), 27.8 (c-d).

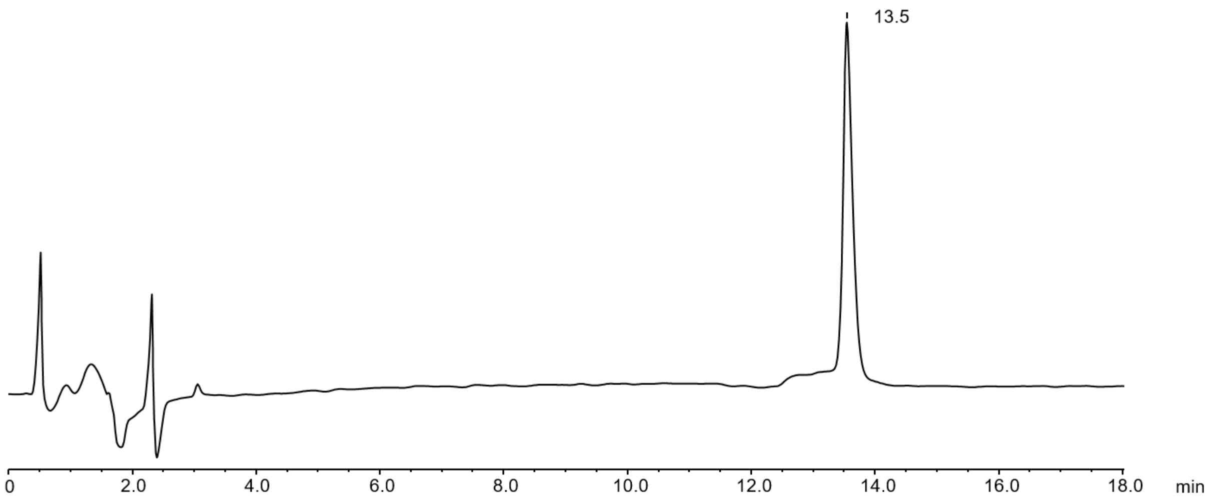

Fig. S13: RP-HPLC chromatogram of T3G3 at λ = 220 nm.

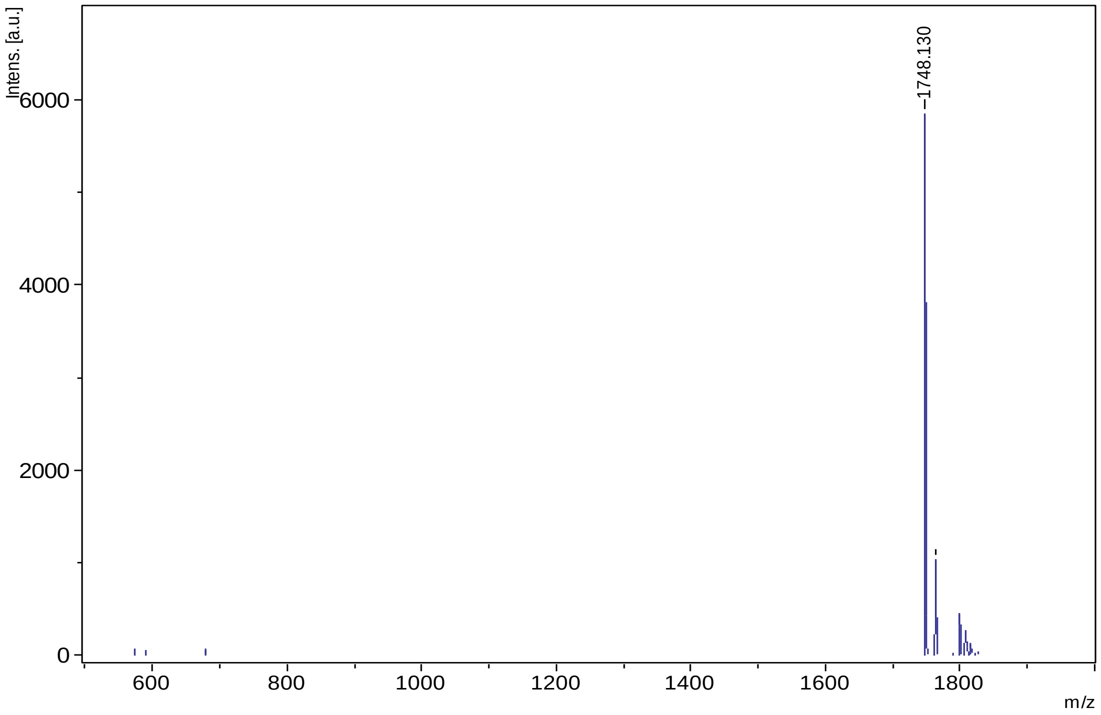

Fig. S14: Mass spectrum of T3G3.

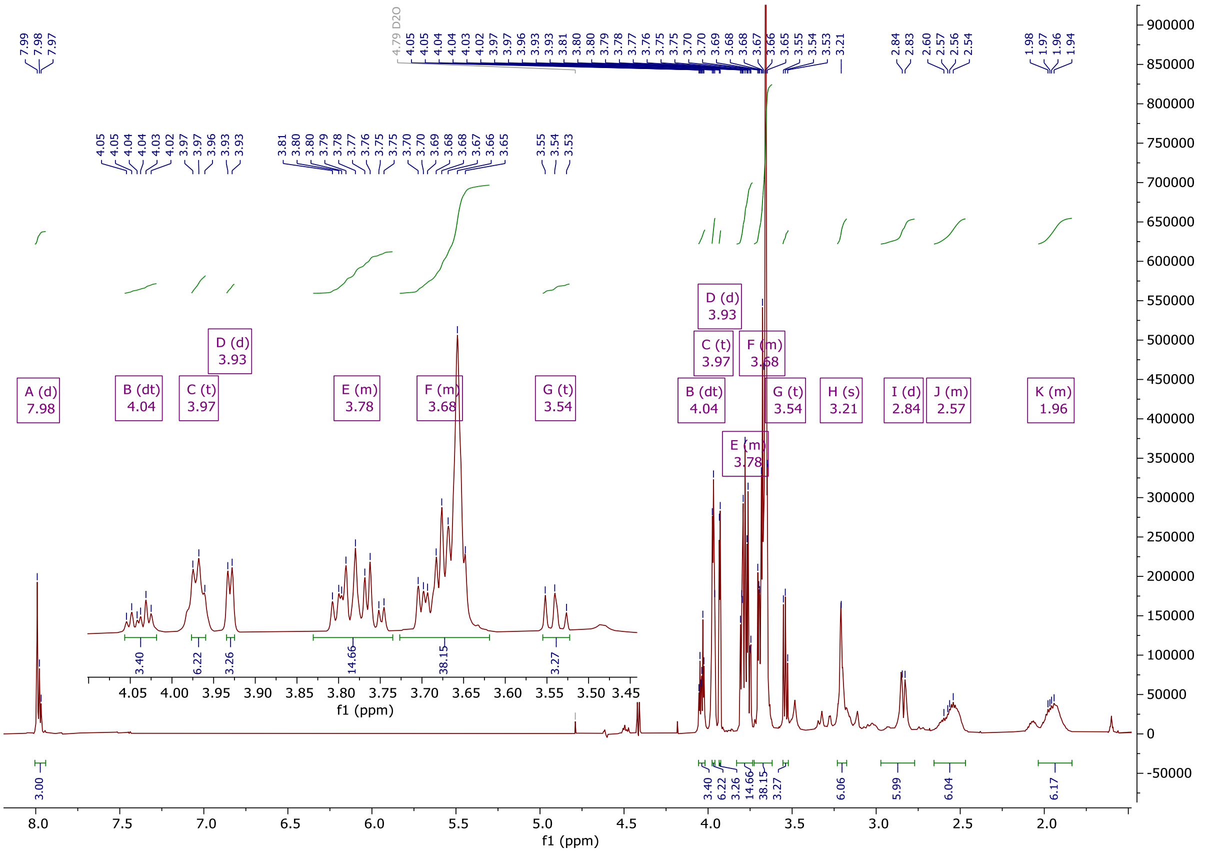

Fig. S15: ^{1}H-NMR spectrum of T3G3.

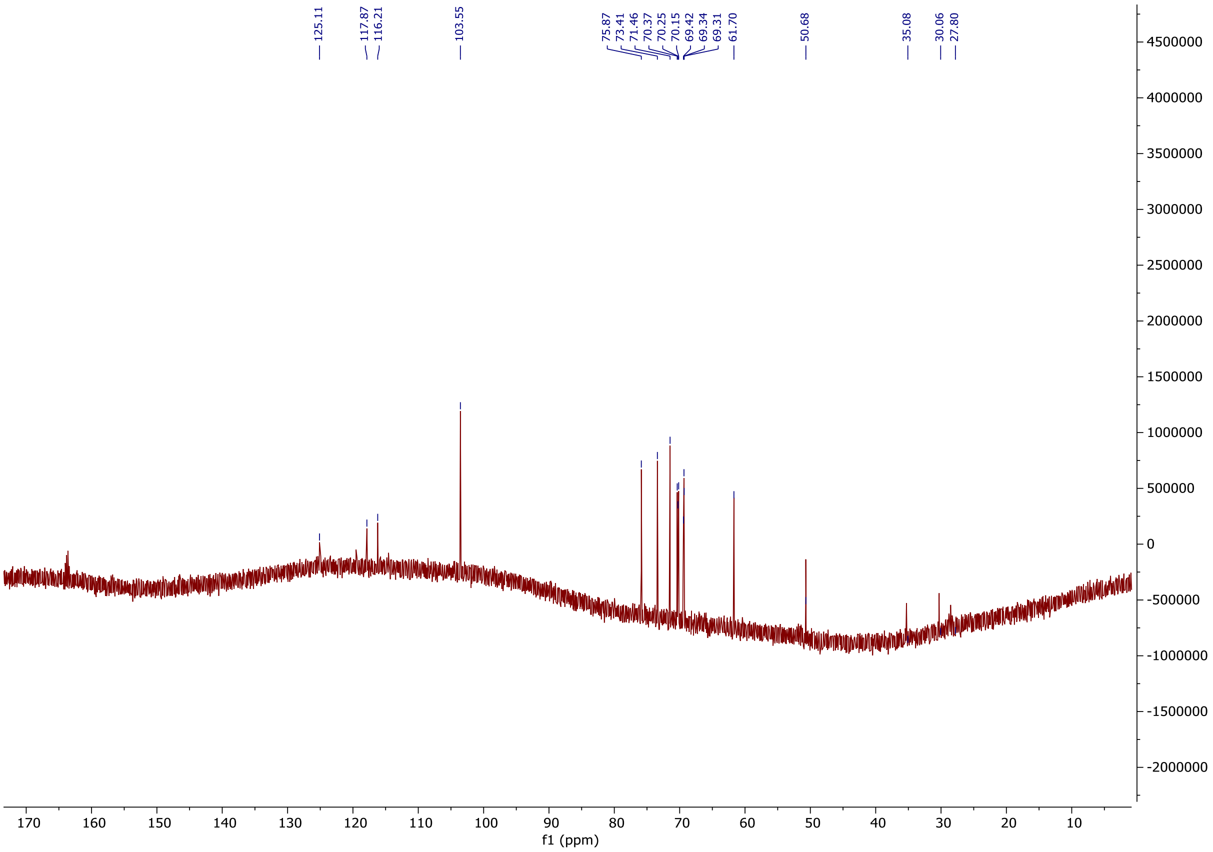

Fig. S16: ^{13}C{^{1}H}-NMR spectrum of T3G3.

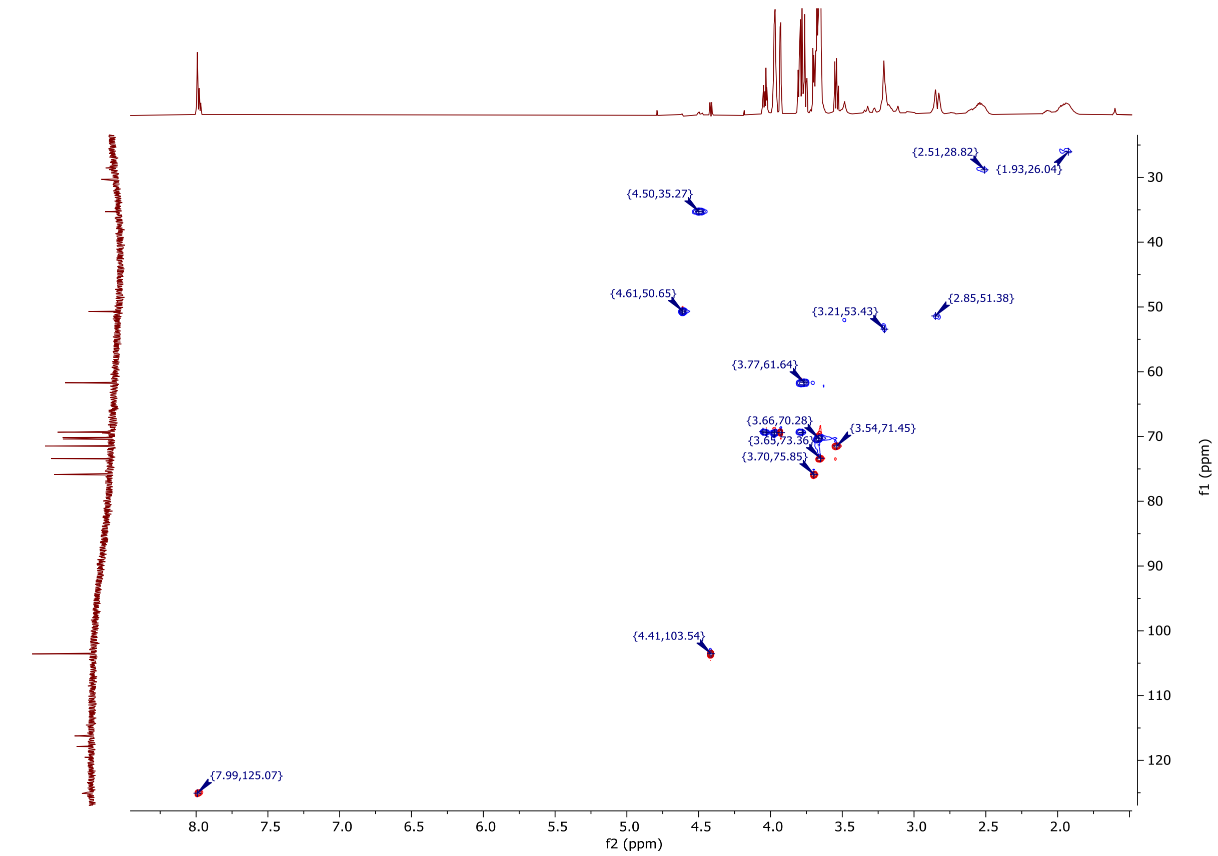

Fig. S17: HSQC-NMR spectrum of T3G3 showing cross peaks between 2.0-8.0 ppm (^{1}H) and 25-125 ppm (^{13}C).

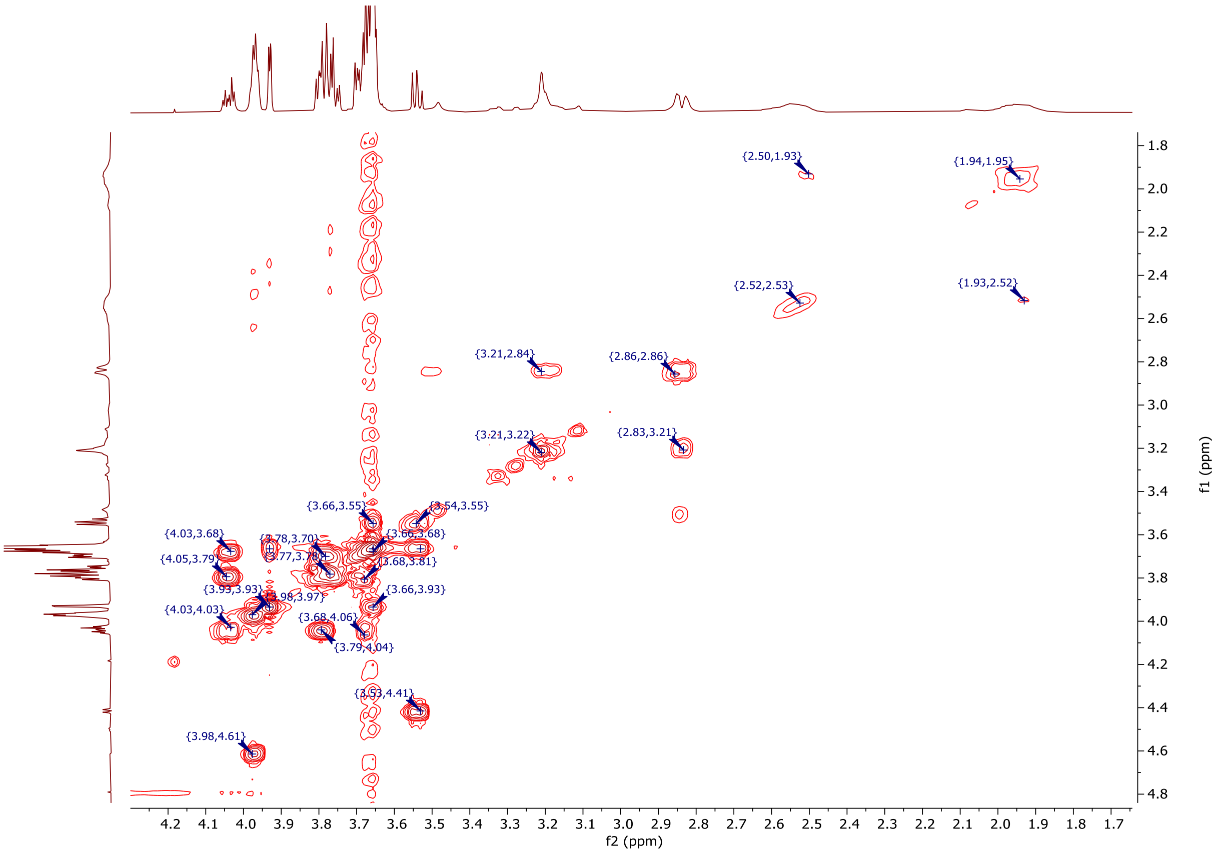

Fig. S18: {^{1}H, ^{1}H}-COSY spectrum of T3G3 showing correlations between 1.8 -4.8 ppm.

T3N3

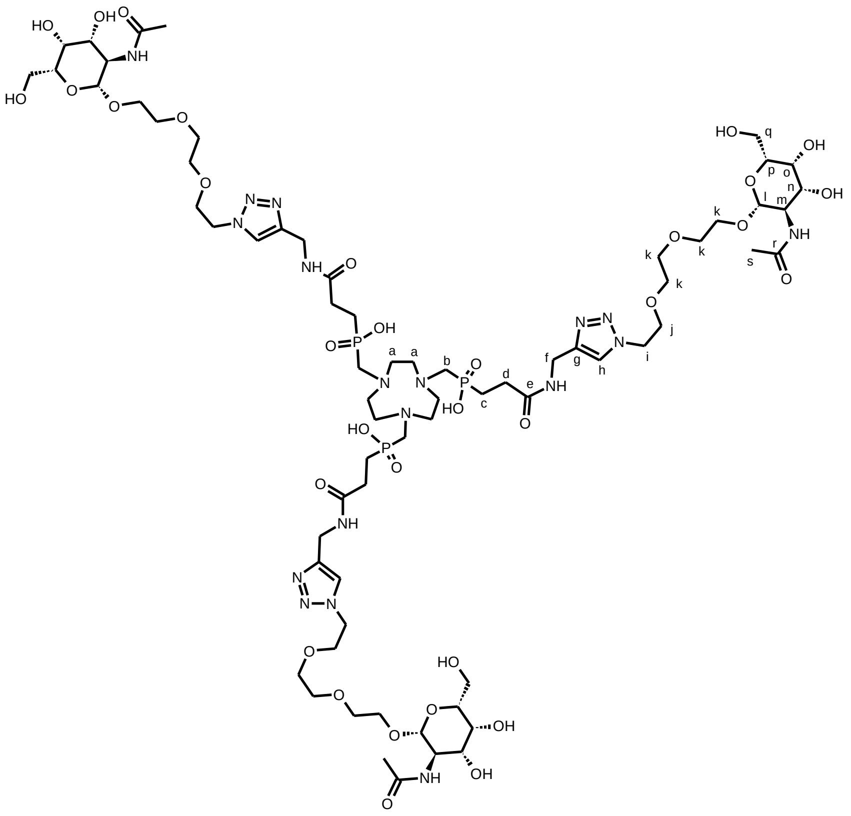

T3N3

Formula Weight: 1825.74 g/mol
Monoisotopic Mass: 1824.77 Da
Molecular Formula: $C_{69}H_{123}N_{18}O_{33}P_3$

TRAP(Alkyne)$_3$ (3.5 mg, 5.0 µmol, 1.0 eq) was dissolved in 100 µL of Millipore water and mixed with a methanolic solution of 2-[2-(2-Azidoethoxy)ethoxy]ethyl 2-acetamido-2-deoxy-β-D-galactopyranoside (6 mg, 15.8 µmol, 3.6 eq). Next, solutions of Cu(OAc)$_2$ (1.0 mg, 5.3 µmol, 1.2 eq) and sodium ascorbate (34.8 mg, 176 mmol, 40.0 eq) in a minimum amount of water were added. This mixture was incubated for 1 hour at 60 °C. For subsequent demetallation, a solution of DTPA (65.6 mg, 30.0 eq) was added and the pH was adjusted to 2.2 using concentrated HCl. After 1 hour at 60 °C the reaction mixture was directly injected into semipreparative HPLC (8-12 % B in 25 min). Lyophilization yielded 880 µg (482 nmol, 10 %) of a colourless solid.

Analytical HPLC (ReproSil Pur, 5-15 % B in 15 min, 1.0 mL/min) t_R = 14.5 min (14 % B).

MALDI-MS (m/z) = 1823.8 $[M-H]^-$, 1885.5 $[M-H+Na+K]^-$.

^{1}H-NMR (600 MHz, D_2O): δ (ppm) = 8.03 (s, 3 H, h), 4.63 (t, ^3J = 7 Hz, 5 H, j), 4.49 (s, 5 H, f), 4.47 (d, ^3J = 8.5 Hz, 3 H, l), 3.97-3.94 (m, 9 H, i/p), 3.92 (d, ^3J = 3 Hz, 3 H, o), 3.89 (dd, ^3J =10.8/8.5 Hz, 3 H, m), 3.81-3.76 (m, 6 H, k), 3.74-3.69 (m, 7 H, n/k), 3.67-3.56 (m, 22 H, q/k), 3.43 (s, 12 H, a), 3.37 (d, ^3J = 5.5 Hz, 6 H, b), 2.56-2.51 (m, 6 H, c), 2.04-2.00 (m, 6 H, d), 1.99 (s, 9 H, s).

^{13}C-NMR (176 MHz, D_2O): δ (ppm) = 175.4 (c-e), 164.0/163.7/163.5 (c-r), 144.6 (c-g), 125.4 (c-h), 102.3 (c-l), 75.8 (c-p), 71.8 (c-n), 70.5 (c-k), 70.3 (c-q), 69.6 (c-k), 69.3 (c-i), 68.5 (c-o), 61.7 (c-k), 55.1/54.5 (c-b), 53.1 (c-m), 52.2 (c-a), 51.2 (c-j), 35.0 (c-f), 28.5 (c-c), 26.4/25.8 (c-d), 22.9 (c-s).

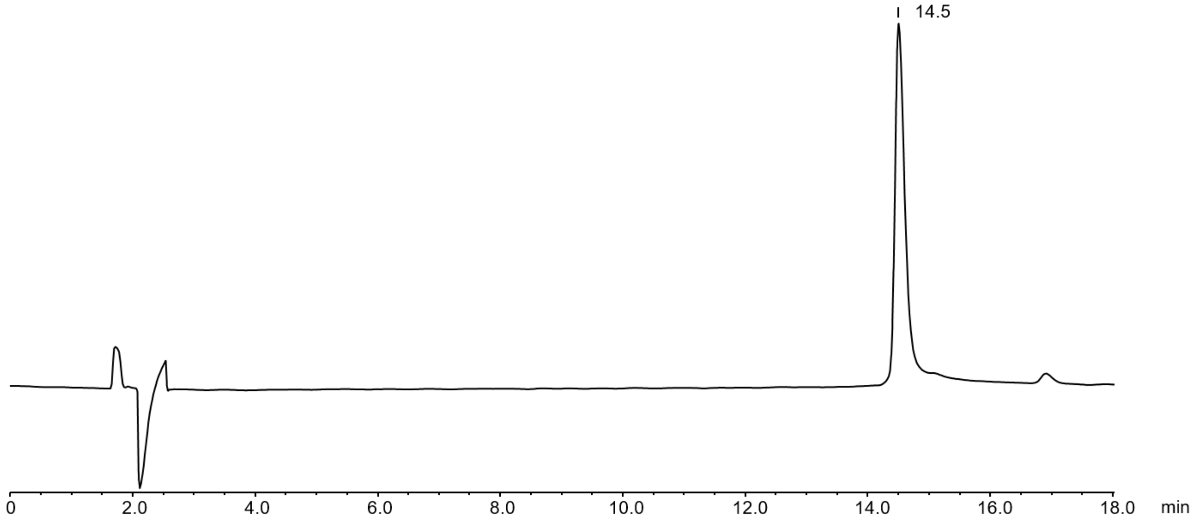

Fig. S19: RP-HPLC Chromatogram of T3N3.

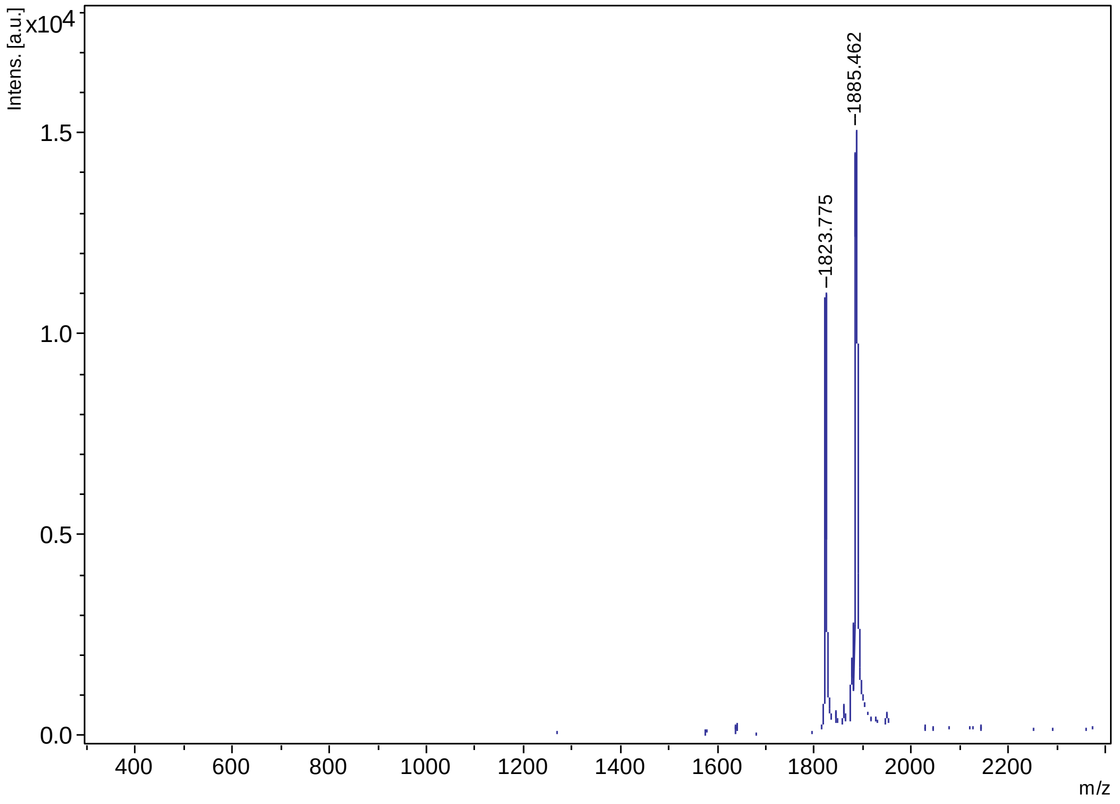

Fig. S20: Mass spectrum of T3N3.

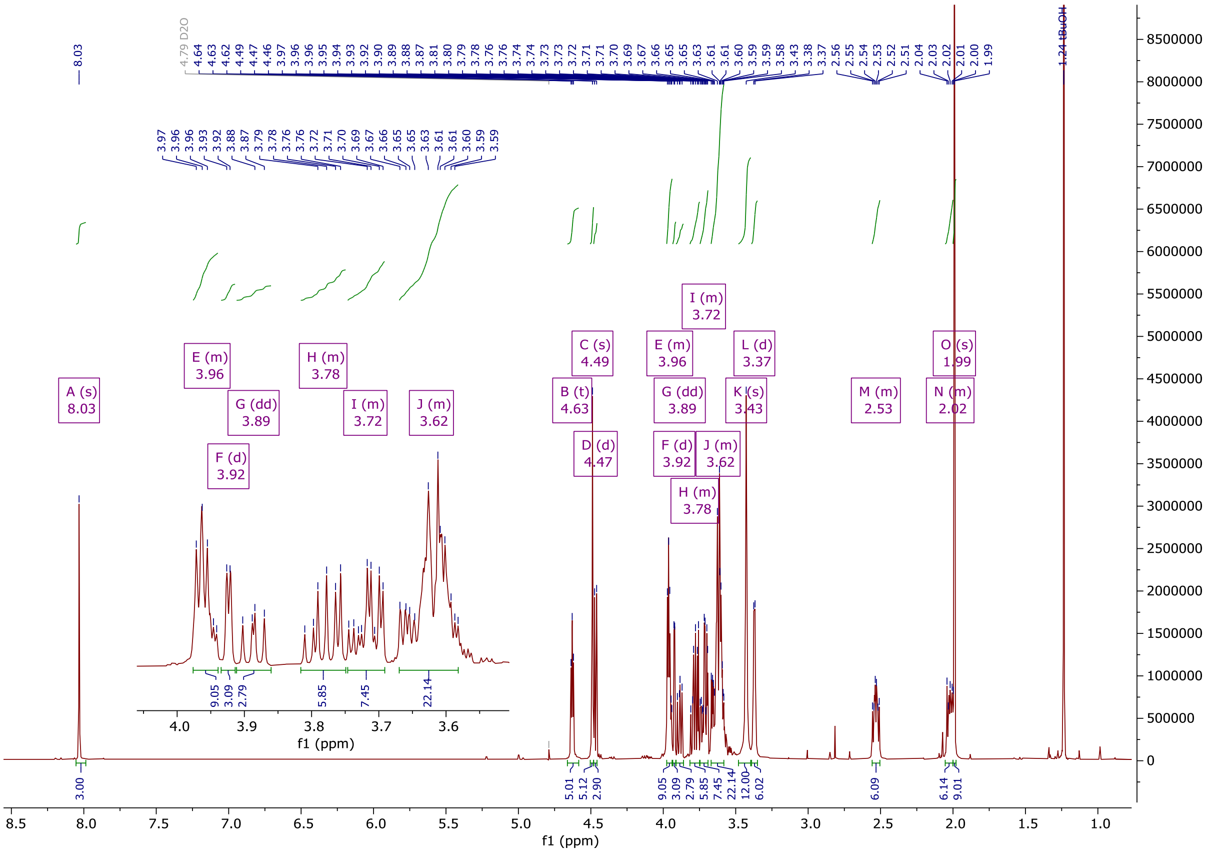

Fig. S21: ^{1}H-NMR spectrum of T3N3.

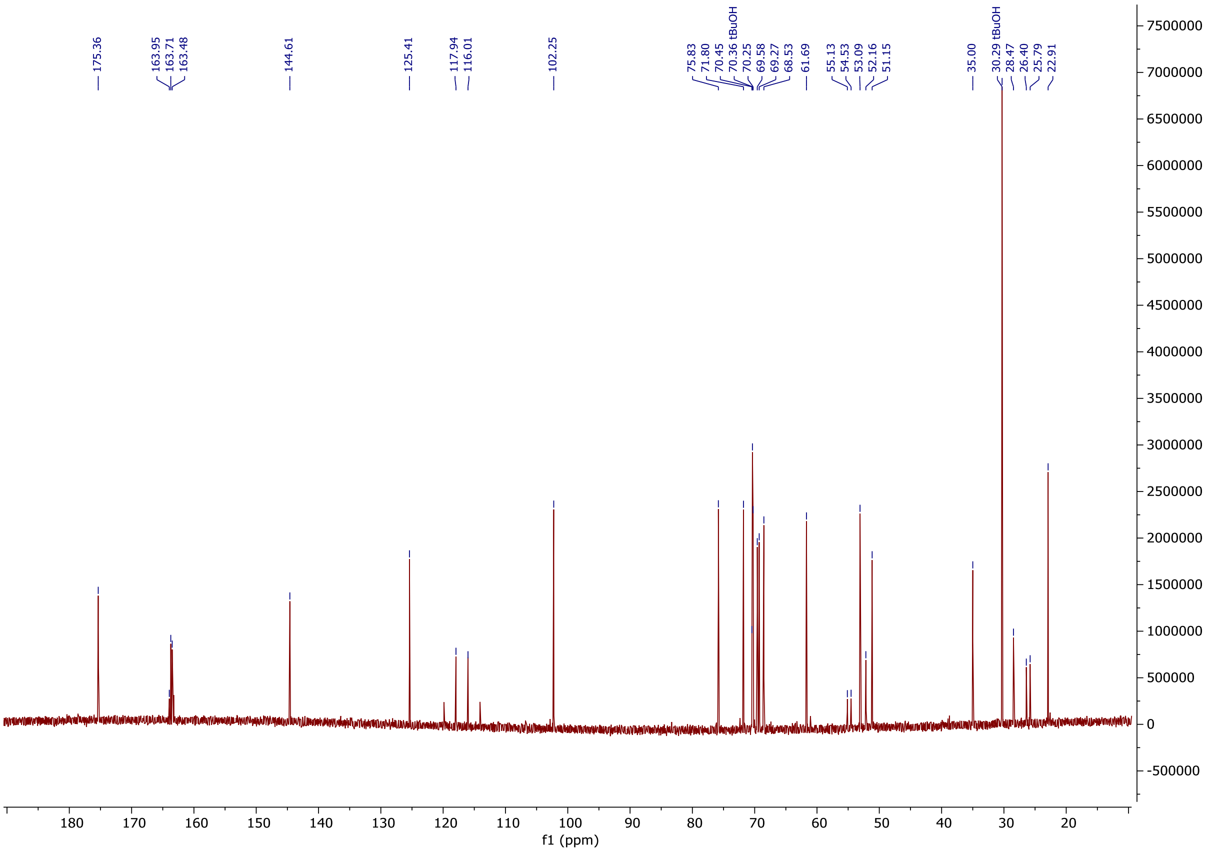

Fig. S22: ^{13}C{^{1}H}-NMR spectrum of T3N3.

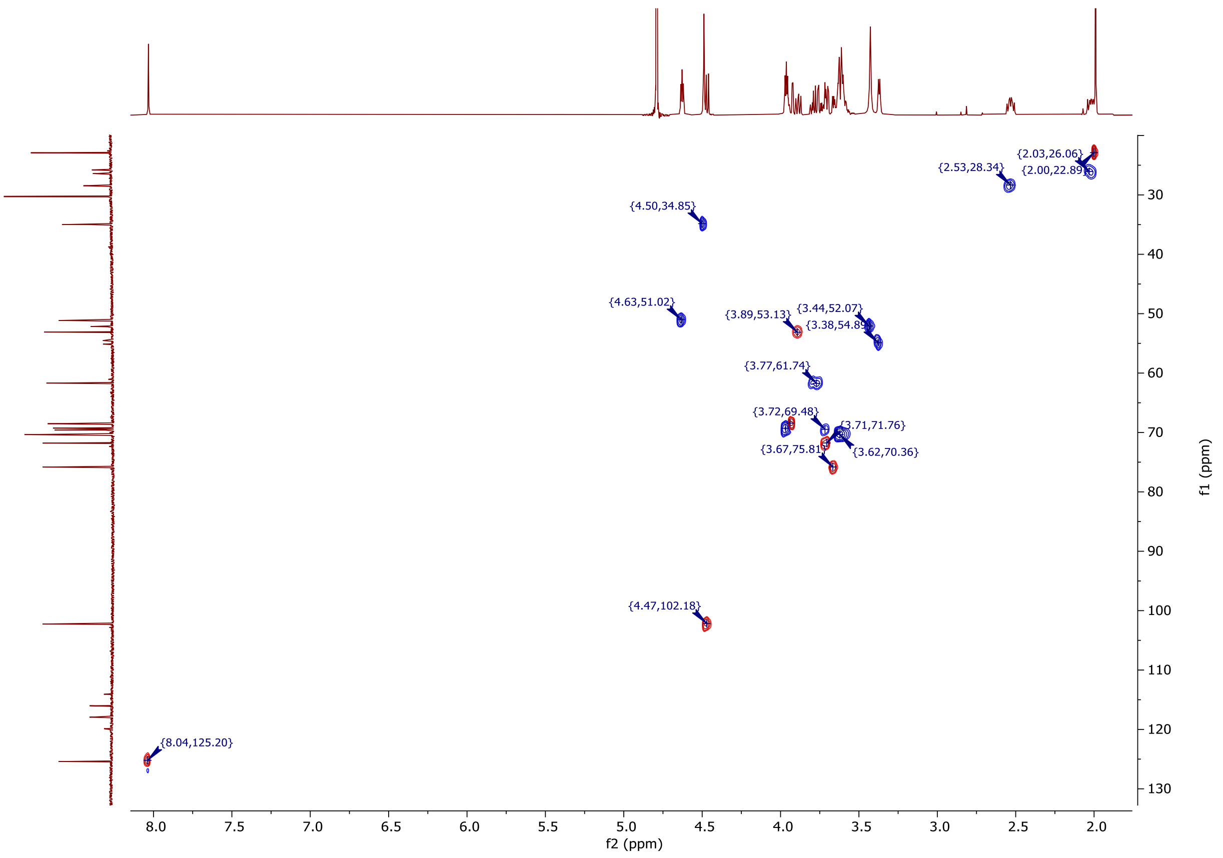

Fig. S23: HSQC-NMR spectrum of T3N3 showing cross peaks between 2.0-8.0 ppm (^{1}H) and 20-130 ppm (^{13}C).

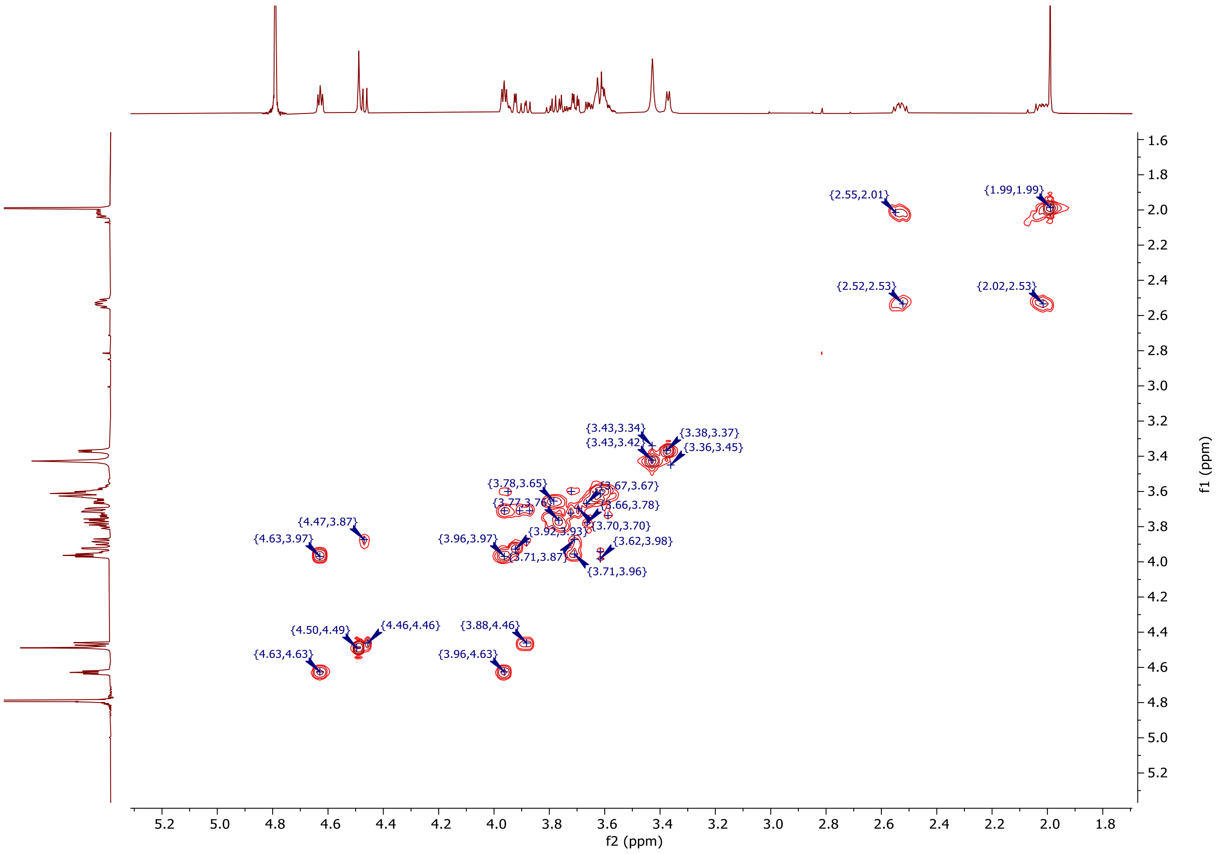

Fig. S24: {^{1}H, ^{1}H}-COSY spectrum of T3N3 showing correlations between 1.6-5.2 ppm.

T3U3

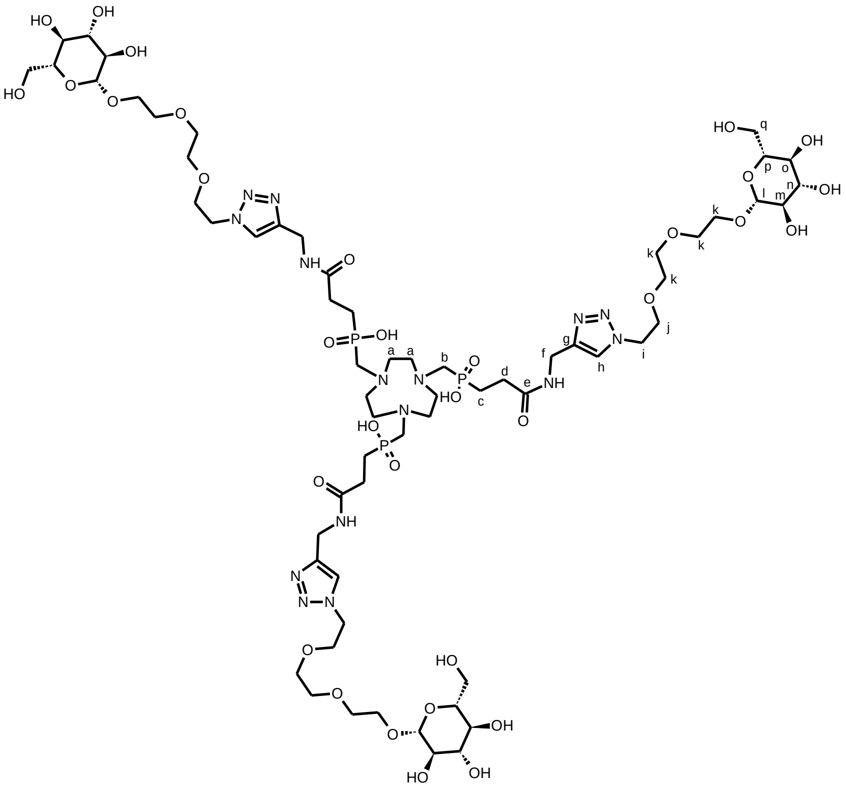

T3U3

Formula Weight: 1702.58 g/mol
Monoisotopic Mass: 1701.69 Da
Molecular Formula: $C_{63}H_{114}N_{15}O_{33}P_3$

TRAP(Alkyne)$_3$ (7.8 mg, 11 µmol, 1.0 eq) was dissolved in 100 µL of Millipore water and mixed with a methanolic solution of 2-[2-(2-Azidoethoxy)ethoxy]ethyl β-D-glucopyranoside (11.8 mg, 35.0 µmol, 3.6 eq). Next, solutions of Cu(OAc)$_2$ (2.3 mg, 11.8 µmol, 1.2 eq) and sodium ascorbate (66 mg, 333 µmol, 34.0 eq) in a minimum amount of water were added. This mixture was incubated for 1 hour at 60 °C. For subsequent demetallation, a solution of DTPA (149 mg, 300 µmol, 30.0 eq) was added and the the pH was adjusted to 2.2 using concentrated HCl. After 1 hour at 60 °C the reaction mixture was directly injected into semipreparative HPLC (8-12 % B in 25 min). Lyophilization yielded 2.4 mg (1.42 µmol, 13 %) of a colourless solid.

Analytical HPLC (ReproSil Pur, 5-15 % B in 15 min, 1.0 mL/min) t_R = 13.6 min (13 % B).

MALDI-MS (m/z) = 678.8 $[2(M-5H)]^{5-}$, 1190.8 $[2(M-H+2Na+K)]^{3-}$, 1703.2 $[M+H]^-$.

^{1}H-NMR (700 MHz, D_2O): δ (ppm) = 7.97 (s, 3 H, h), 4.61 (t, ^3J = 5.0 Hz, 5 H, i), 4.47 (t, ^3J = 4.0 Hz, 9 H, f/l), 4.02 (dt, ^3J = 11.4/4.2 Hz, 3 H, k), 3.97 (t, ^3J = 5.1 Hz, 7 H, j), 3.92 (dd, 12.4/2.2 Hz, 3 H, q), 3.79 (dt, ^3J = 11.5/4.7 Hz, 3 H, k), 3.72 (dd, ^3J = 12.3/6.0 Hz, 3 H, q), 3.67-3.65 (m, 18 H, k), 3.51-3.44 (m, 18 H, a/n/p), 3.39 (t, ^3J = 9.4 Hz, 3 H, o), 3.31-3.28 (m, 9 H, b/m), 2.53-2.49 (m, 6 H, c), 1.98-1.93 (m, 6 H, d).

^{13}C-NMR (176 MHz, D_2O): δ (ppm) = 175.6 (c-e), 145.2 (c-g), 125.0 (c-h), 102.9 (c-l), 76.6 (c-p), 76.4 (c-n), 73.8 (c-m), 70.3 (c-k), 70.2 (c-o), 69.4 (c-j/c-k), 61.4 (c-q), 55.3 (c-b), 52.1 (c-a), 50.7 (c-i), 35.3 (c-f), 28.8 (c-c), 27.2 (c-d).

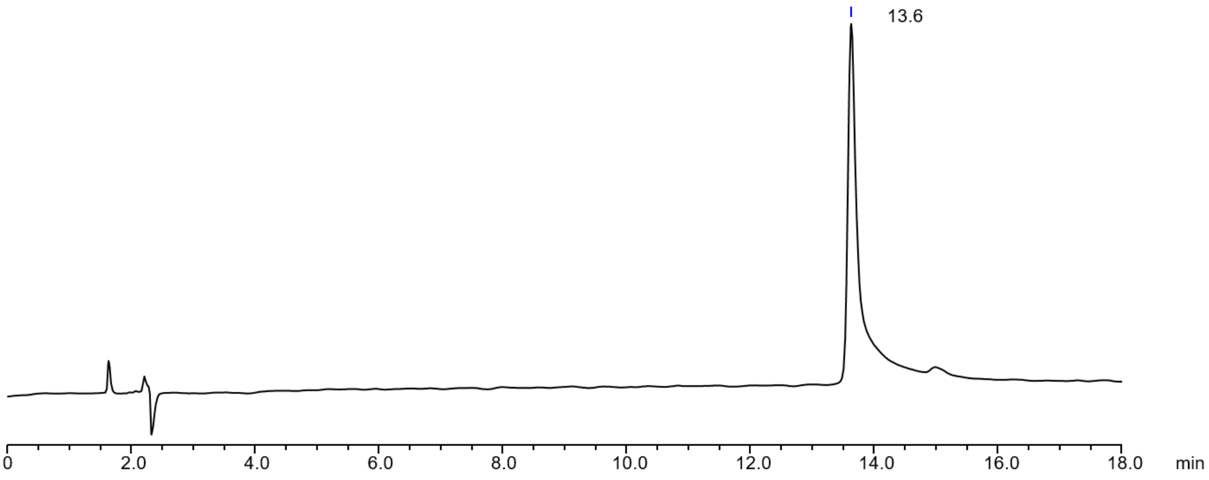

Fig. S25: RP-HPLC chromatogram of T3U3 at λ = 220 nm.

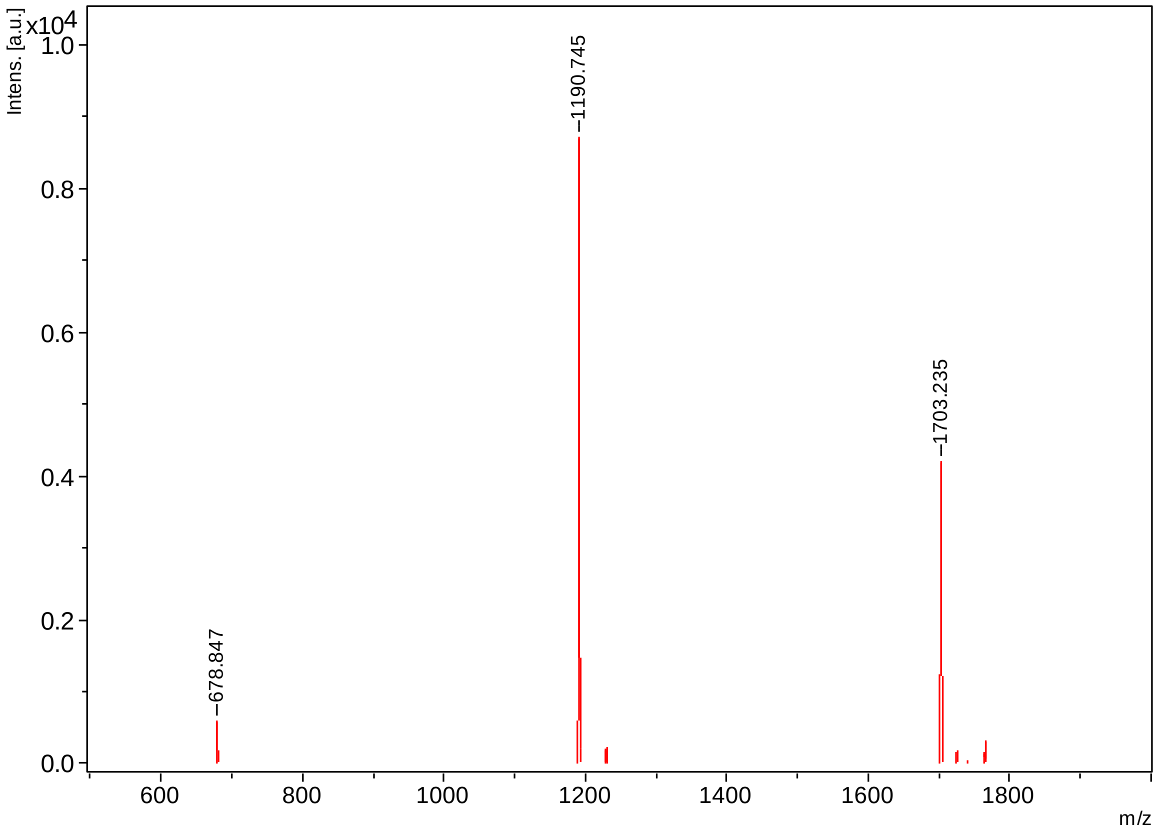

Fig. S26: Mass spectrum of T3U3.

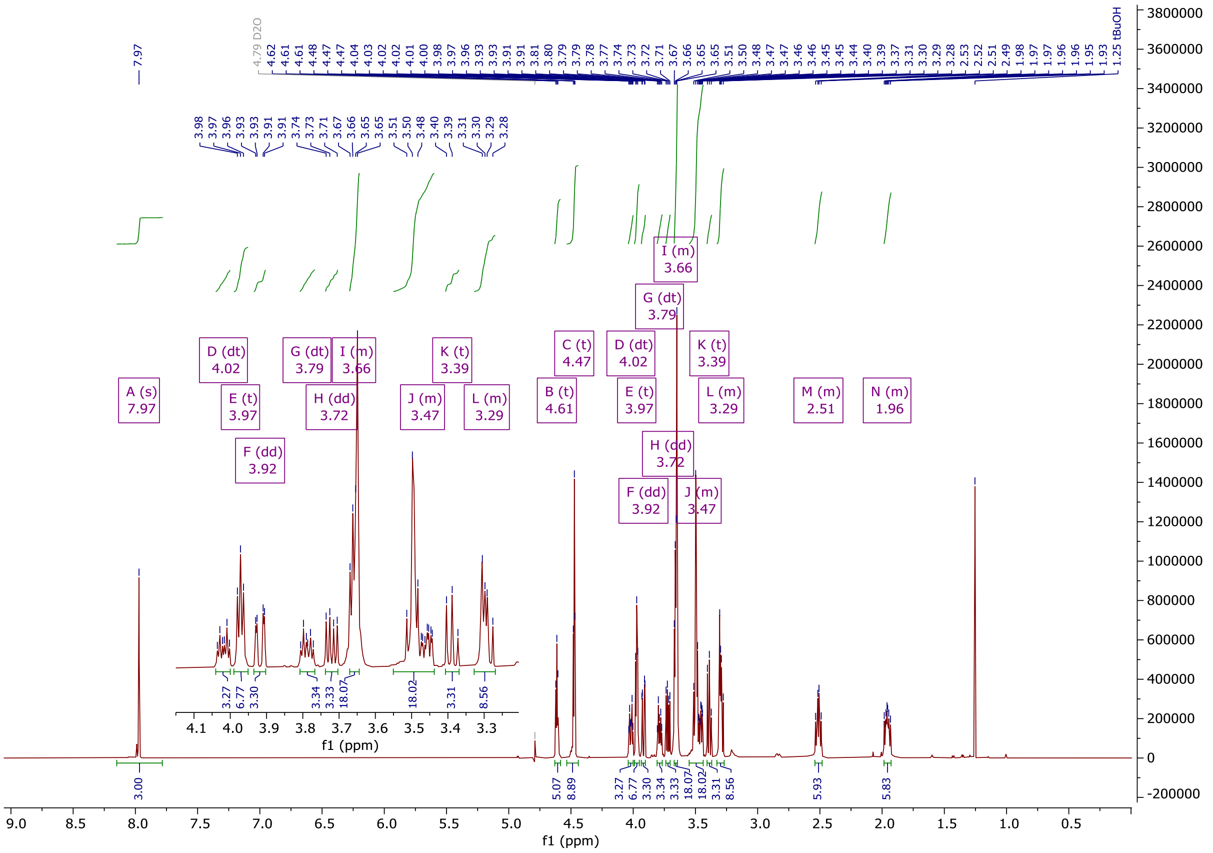

Fig. S27: ^{1}H-NMR spectrum of T3U3.

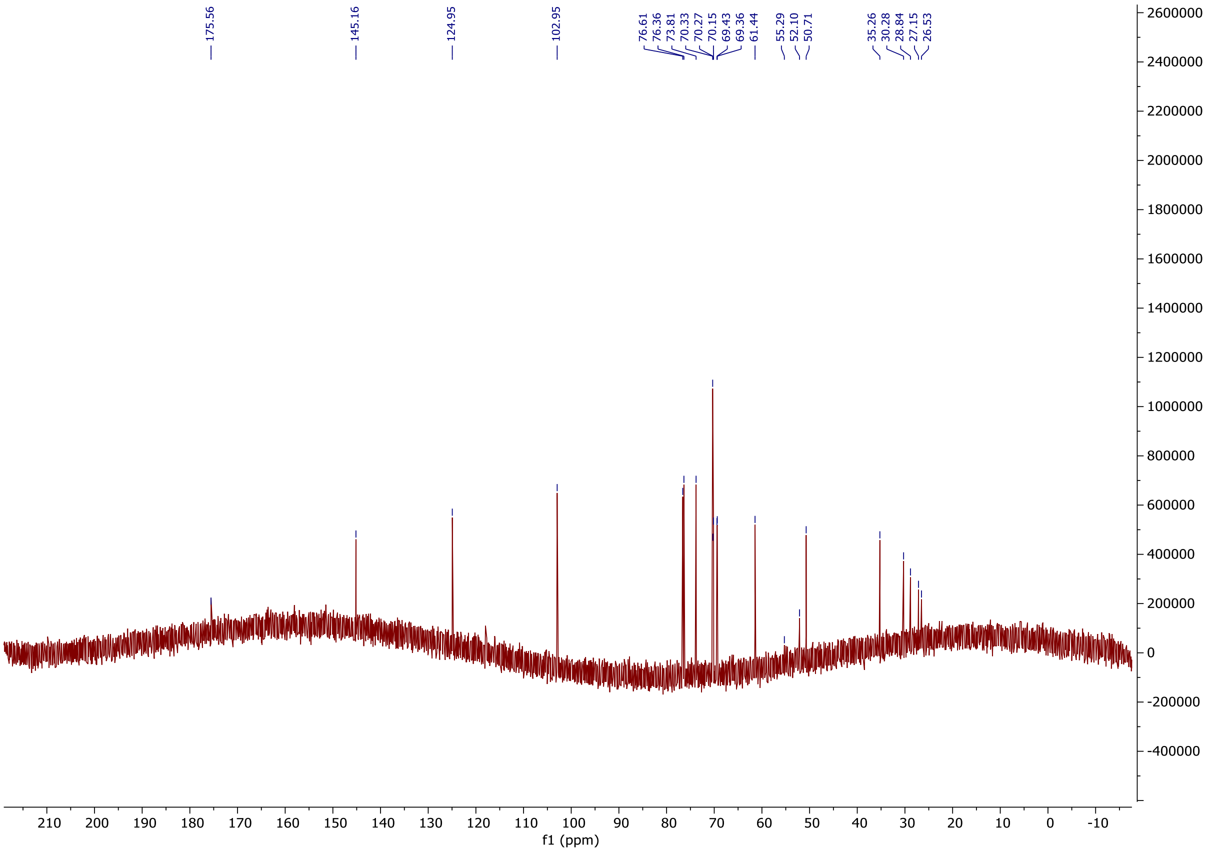

Fig. S28: ^{13}C{^{1}H}-NMR spectrum of T3U3.

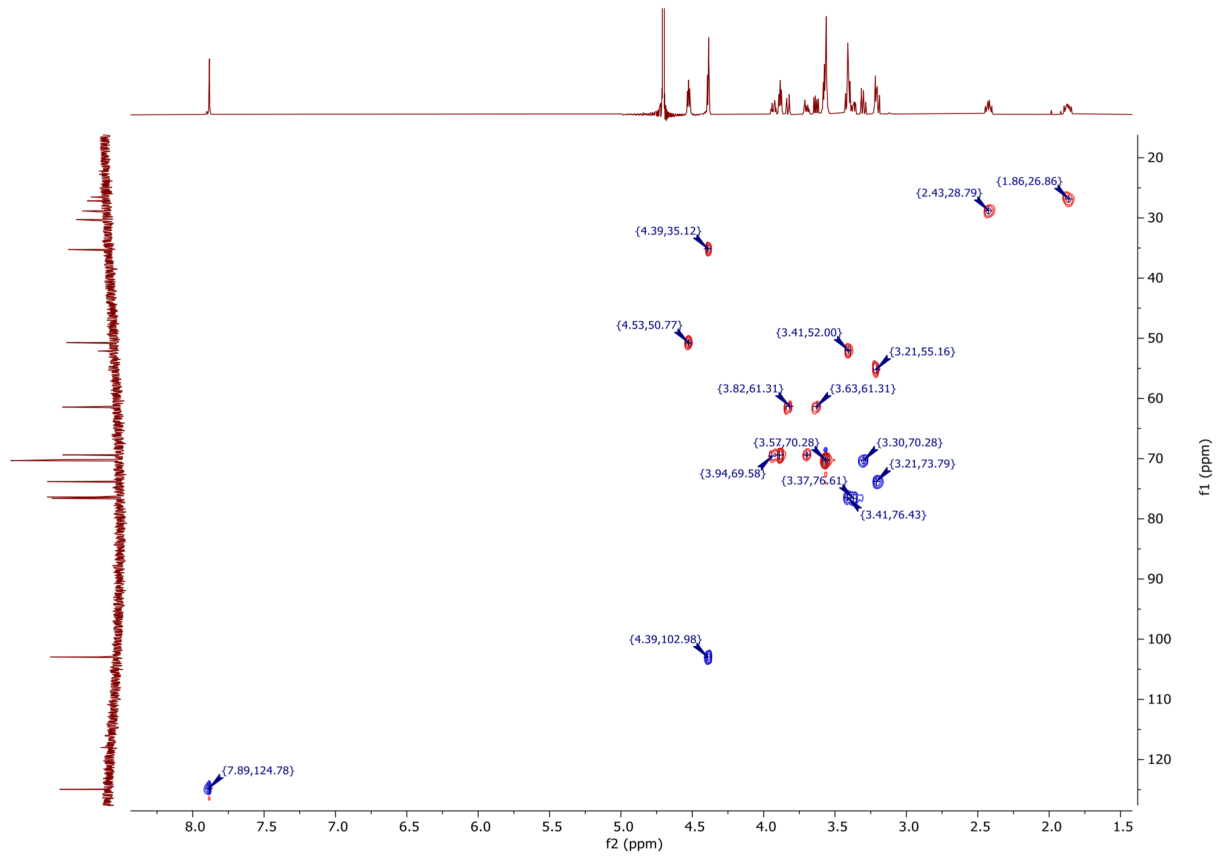

Fig. S29: HSQC-NMR spectrum of T3U3 showing cross peaks between 1.5-8.0 ppm (^{1}H) and 20-120 ppm (^{13}C).

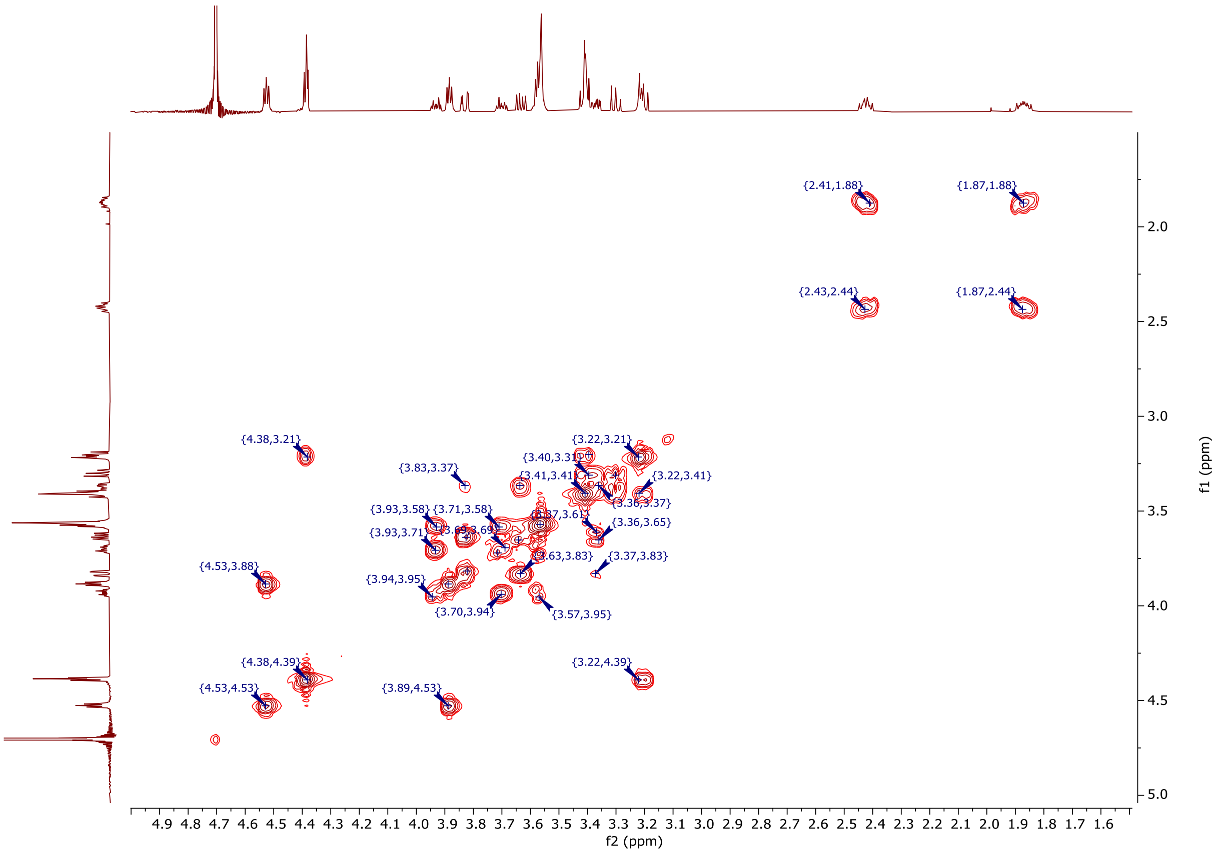

Fig. S30: {^{1}H, ^{1}H}-COSY spectrum of T3N3 showing correlations between 1.5-5.0 ppm.

Solid-Phase Binding Assay

Preparation of [^{125}I]I-Asiaoloorosomucoid (ASOR), which serves as the radioactive standard ligand for the affinity assay, followed a previously published procedure (1).

In a flat-bottom Nunc® Maxisorp™ 96 well plate (Thermofisher, Waltham, Massachusetts, USA) human recombinant ASGR1 (#4394-AS, R&D Systems, Minneapolis, USA) was immobilized overnight in the fridge at a concentration of 1 µg/mL in 100 µL of carbonate buffer (15 mM Na_2CO_3, 35 mM $NaHCO_3$, pH 9.6). The coating buffer was discarded and the wells were washed three times with 200 µL of TRIS-buffered saline (TBS; 20 mM TRIS + 150 mM NaCl + 2 mM $CaCl_2$ + 0.05 % TWEEN 20, pH 7.4). For blocking of unspecific binding wells were incubated for 1 hour at room temperature with 150 µL of TBS+BSA (20 mM TRIS + 300 mM NaCl + 4 mM $CaCl_2$ + 1 % BSA, pH 7.4) under gentle agitation (300 rpm). Then, wells were washed again with TBS (3 × 200 µL) and the plate was quickly dried by knocking it upside down on a paper tissue. For the assay 80 µL of TBS were transferred into each well, followed by the addition of 10 µL of a dilution series (10^{-4}-10^{-10} M) of cold complex in PBS + 0.1 % BSA as well as 10 µL of a 50 nM ^{125}I-ASOR solution in PBS (~150 000 cpm/well). Thus, final concentrations were ranging from 10^{-5}-10^{-11} M. The plate was incubated for 1 hour at room temperature under mild agitation (300 rpm). Supernatants were removed and wells were washed twice with 200 µL of TBS. Supernatant and wash fractions were combined in a reagent tube, representing the free radioligand. For the release of surface-bound activity, wells were incubated for 10 min with 200 µL of hot (60 °C) 1 M NaOH. Lysates were removed and wells were washed twice with 200 µL of 1 M NaOH. Lysates and wash fractions were collected in a separate reagent tube and the activity content was measured in a γ-counter. Values were plotted in Excel and fitting of the sigmoidal binding curve was achieved using Excel's solver plugin.

The presence of the ASGR1 can be confirmed by adding 100 µL (1.0 µg/mL) of a murine anti-human ASGR1 antibody instead of the dilution series. After 1 hour of incubation at room temperature (300 rpm), the wells were washed with TBS (3 × 200 µL) and 100 µL of a horseradish peroxidase-coupled anti-mouse antibody (1:500 dilution) (#HAF007, R&D Systems) were added. Wells were incubated for 1 hour at room temperature (300 rpm) followed by another washing step with TBS (3 × 200 µL). For read-out, 50 µL of Seramun Blau Fast 2 Horseradish Peroxidase Substrate (Seramun Diagnostica GmbH, Heidesee, Germany) was added to the wells and the reaction was allowed to progress for 5 min in the dark. Colour development was stopped by the addition of 50 µL 3 M H_2SO_4 and the absorbance at 450 nm was measured with a plate reader.

Equilibrium studies on TRAP-Conformers

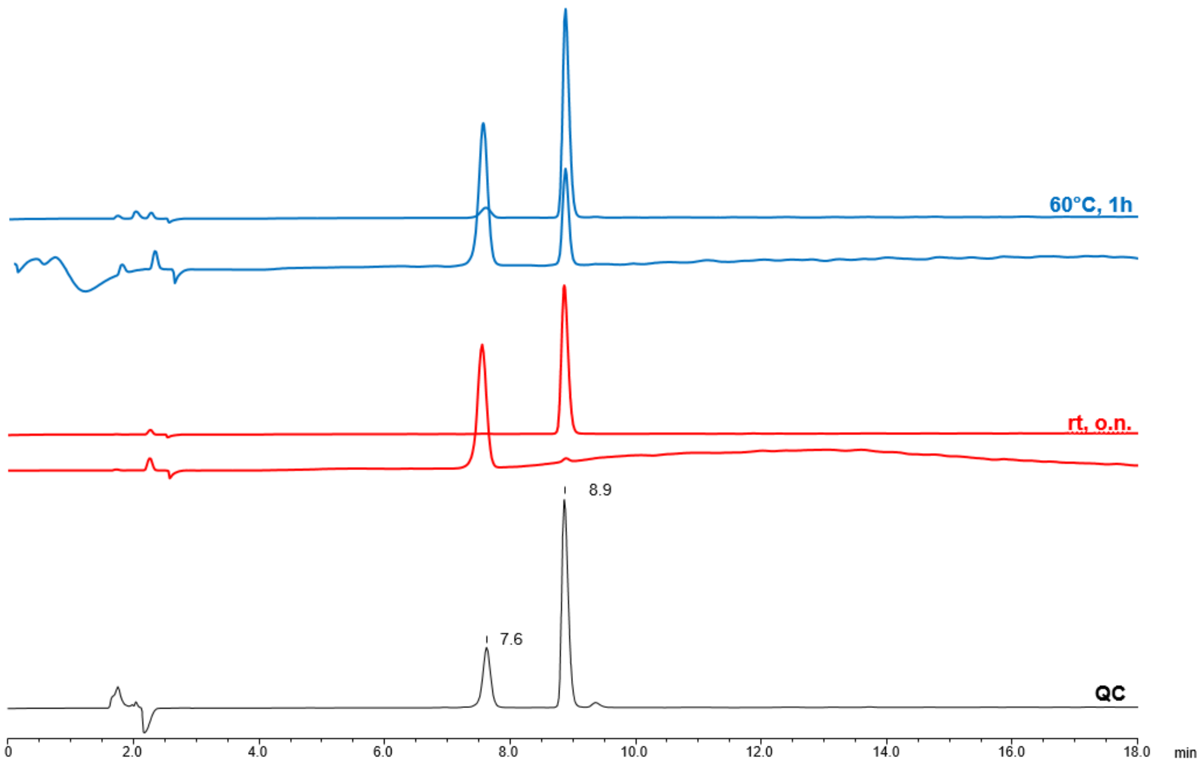

Fig. S31: Exemplary analytical HPLC traces of metal-induced TRAP-conformers upon chelation of ^{nat}Ga at λ = 220 nm; **black:** quality control of ^{nat}Ga-T0G3; **red:** reinjection of isolated peaks after incubation at room temperature overnight; **blue:** reinjection of isolated peaks after heating to 60 °C for 1 hour (Column: ReproSil Pur C_{18} AQ 150 × 4.6 mm, 5 µm, 120 Å, Gradient: 1-10 % MeCN + 0.1 % TFA in 15 min, Flow: 1 mL/min).

Literature

(1) Zierke MA, Rangger C, Samadikhah K, Panzer M, Dichtl S, Hörmann N, Wilflingseder D, Schmid AM, Haubner R. [^{68}Ga]Ga-NODAGA-TriGalactan, a low molecular weight Tracer for the non-invasive Imaging of the functional Liver Reserve. *EJNMMI Radiopharmacy and Chemistry*. **2024**, 9 (1), 41.
